# Spatiotemporal diversity in molecular and functional abnormalities in the mdx dystrophic brain

**DOI:** 10.1186/s10020-025-01109-5

**Published:** 2025-03-20

**Authors:** Joanna Pomeroy, Malgorzata Borczyk, Maria Kawalec, Jacek Hajto, Emma Carlson, Samuel Svärd, Suraj Verma, Eric Bareke, Anna Boratyńska-Jasińska, Dorota Dymkowska, Alvaro Mellado-Ibáñez, David Laight, Krzysztof Zabłocki, Annalisa Occhipinti, Loydie Majewska, Claudio Angione, Jacek Majewski, Gennady G. Yegutkin, Michal Korostynski, Barbara Zabłocka, Dariusz C. Górecki

**Affiliations:** 1 School of Medicine, Pharmacy and Biomedical Sciences, University of Portsmouth, White Swan Road, Portsmouth, PO1 2DT UK; 2 Department of Molecular Neuropharmacology, Maj Institute of Pharmacology, Polish Academy of Sciences, 12 Smętna Str., 31-343 Krakow, Poland; 3 Molecular Biology Unit, Mossakowski Medical Research Institute, Polish Academy of Sciences, Warsaw, Poland; 4 Department of Human Genetics, McGill University, Montreal, QC H3A 1B1 Canada; 5 MediCity Research Laboratory and InFLAMES Flagship, University of Turku, Turku, Finland; 6 School of Computing, Engineering and Digital Technologies, Teesside University, Middlesbrough, UK; 7 Laboratory of Cellular Metabolism, Nencki Institute of Experimental Biology, Warsaw, Poland; 8 Department of Pediatrics, McGill University, McGill Health Centre Glen Site, 1001 Decarie Blvd, EM02210, Montreal, QC H4A 3J1 Canada

## Abstract

Duchenne muscular dystrophy (DMD) is characterized by progressive muscle degeneration and neuropsychiatric abnormalities. Loss of full-length dystrophins is both necessary and sufficient to initiate DMD. These isoforms are expressed in the hippocampus, cerebral cortex (Dp427c), and cerebellar Purkinje cells (Dp427p). However, our understanding of the consequences of their absence, which is crucial for developing targeted interventions, remains inadequate. We combined RNA sequencing with genome-scale metabolic modelling (GSMM), immunodetection, and mitochondrial assays to investigate dystrophic alterations in the brains of the mdx mouse model of DMD. The cerebra and cerebella were analysed separately to discern the roles of Dp427c and Dp427p, respectively. Investigating these regions at 10 days (10d) and 10 weeks (10w) followed the evolution of abnormalities from development to early adulthood. These time points also encompass periods before onset and during muscle inflammation, enabling assessment of the potential damage caused by inflammatory mediators crossing the dystrophic blood–brain barrier. For the first time, we demonstrated that transcriptomic and functional dystrophic alterations are unique to the cerebra and cerebella and vary substantially between 10d and 10w. The common anomalies involved altered numbers of retained introns and spliced exons across mdx transcripts, corresponding with alterations in the mRNA processing pathways. Abnormalities in the cerebra were significantly more pronounced in younger mice. The top enriched pathways included those related to metabolism, mRNA processing, and neuronal development. GSMM indicated dysregulation of glucose metabolism, which corresponded with GLUT1 protein downregulation. The cerebellar dystrophic transcriptome, while significantly altered, showed an opposite trajectory to that of the cerebra, with few changes identified at 10 days. These late defects are specific and indicate an impact on the functional maturation of the cerebella that occurs postnatally. Although no classical neuroinflammation markers or microglial activation were detected at 10 weeks, specific differences indicate that inflammation impacts DMD brains. Importantly, some dystrophic alterations occur late and may therefore be amenable to therapeutic intervention, offering potential avenues for mitigating DMD-related neuropsychiatric defects.

**Supplementary Information:**

The online version contains supplementary material available at 10.1186/s10020-025-01109-5.

## Introduction

Duchenne muscular dystrophy (DMD) is a neuromuscular disorder associated with varying degrees of cognitive and behavioural abnormalities (Allen and Rodgin 1960; Anderson et al. 2002; Doorenweerd et al. 2017; Murphy et al. 2015), which are pleiotropic effects of the mutation. DMD patients have an IQ of at least 1 standard deviation lower than that of the unaffected population, with 30% having an IQ < 70 (Allen and Rodgin 1960). Patients present with impairments in short-term and working memory (Cyrulnik and Hinton 2008; Hinton et al. 2007), with delayed onset of speech and abnormal language processing, including impaired phonological processing (Chieffo et al. 2022; Cyrulnik and Hinton 2008; Hinton et al. 2007). A startle response (Maresh et al. 2023) and increased incidences of attention deficit hyperactivity disorder (ADHD), autism spectrum disorder (ASD), and epilepsy (Lionarons et al. 2019; Pane et al. 2013; Ricotti et al. 2016) have also been observed.

DMD is caused by mutations in the *DMD* gene encoding eight dystrophin isoforms. Among all tissues, the brain expresses the greatest variety of *DMD* transcripts, which are specific to particular regions and cell types (Górecki et al. 1992). Of the three full-length 427 kDa dystrophins, two are expressed in specific brain areas: Dp427c, which is particularly abundant in the hippocampal Amon’s horn, cerebral cortex and amygdala, and Dp427p, which is specifically expressed in cerebellar Purkinje cells (Abdulrazzak et al. 2001; Górecki et al. 1992).

The loss of full-length dystrophin expression is both necessary and sufficient to initiate DMD, leading to progressive muscle degeneration and wasting accompanied by severe sterile inflammation (Chen et al. 2005). The dystrophin-null muscle phenotype, although rare and poorly characterized, appears to be exacerbated in both humans and mice (Chesshyre et al. 2022; Young et al. 2020). Neuropsychiatric abnormalities may also worsen when mutations affect short dystrophin isoforms (Daoud et al. 2009; Doorenweerd et al. 2017; Taylor et al. 2010) but the primary molecular defect underlying brain alterations is the loss of full-length dystrophins (Allen and Rodgin 1960).

The mdx mouse is the most widely used animal model of DMD and has a stop mutation in exon 23 of the *DMD* gene (Sicinski et al. 1989), eliminating full-length dystrophin translation. Hence, the mdx molecular defect matches that found in most DMD patients. At 3 to 10 weeks, the mdx phenotype also closely resembles that of the human disease (Duddy et al. 2015; Massopust et al. 2020), including cognitive and behavioural abnormalities. Histologically detectable muscle inflammation, which is a hallmark feature of dystrophic pathology across species, starts in mdx mice at approximately 12 days of age (Disatnik et al. 1998).

Given its chronic nature, the inflammatory mediators released into the bloodstream from the dystrophic muscle affect distant tissues. Importantly, these inflammatory mediators should easily cross the mdx blood‒brain barrier (BBB), which appears permeable (Frigeri et al. 2001; Nico et al. 2003, 2004). Thus, the brain phenotype can be a combination of two abnormalities. The main issue is the lack of dystrophins in developing brain cells, as suggested by the largely nonprogressive nature of this dystrophic abnormality (Tyagi et al. 2019). This leads to structural disarrangements, as exemplified by GABAergic abnormalities in the hippocampal and cerebellar Purkinje neurons of mdx mice and humans (Brünig et al. 2002; Knuesel et al. 1999; Krasowska et al. 2014; Suzuki et al. 2017; Zarrouki et al. 2022). This intrinsic defect can be exacerbated by neuroinflammation fuelled by inflammatory mediators that easily access the brain. The latter mechanism may involve, among others, alterations in the kynurenine pathway (Copeland et al. 2022) and in extracellular ATP signalling. In the latter, ablation of P2X7, the key inducer of inflammation, improved the brain phenotype in mdx mice (Sinadinos et al. 2015).

The identified defects do not account for all neuropsychological abnormalities, and understanding the precise contributions of various mechanisms is crucial for devising effective treatments. This is particularly important considering that severe impairments in brain function affect one-third of patients, making its impact even more severely debilitating.

We investigated, in an unbiased manner, which changes in the dystrophic brain that occur prenatally are still present in adulthood. Moreover, whether abnormalities undergo modifications as the brain matures and whether these abnormalities are further exacerbated by inflammatory mediators permeating through the leaky BBB remain unclear. For this purpose, we used a combination of transcriptomics and functional analyses of specific brain regions at distinct timepoints.

Specifically, 10-day-old and 10-week-old mouse brains were used to distinguish intrinsic developmental abnormalities from those confounded by inflammation. At 10 days, there is yet no significant inflammation in the muscles of the mdx mice, while at 10 weeks, the muscles sustained numerous cycles of degeneration-regeneration with severe inflammation.

Considering the complex expression pattern of full-length dystrophins, with Dp427c present in the hippocampal Amon’s horn, cerebral cortex, and amygdala in the cerebrum and Dp427p in the cerebellar Purkinje neurons only, we analysed these brain regions separately. In this way, we compared the potentially distinct impact of the loss of these two isoforms, which, albeit structurally highly similar, show tightly controlled expression. Notably, both the cerebrum and cerebellum are involved in working memory, language processing, verbal fluency, and executive functions (Frings et al. 2006; Lie et al. 2006), all of which are affected in DMD.

## Materials and methods

A graphical overview of the experimental procedures performed is presented in Supplementary Fig. 1.

### Animals

10 days and 10 weeks old C57BL/10 wild-type and *DMD*^*mdx*^ (mdx) male mice were kept in a pathogen-free environment, fed a standard chow diet and given water ad libitum. They were housed in a 12-h light/dark cycle, 19–23 ℃, and 45–65% humidity. Brain samples were collected at the same time in the morning to eliminate any potential circadian rhythm differences. The study is reported in accordance with the ARRIVE guidelines (https://arriveguidelines.org).

### RNA sequencing

Total RNA was extracted from the cerebra and cerebella of both age groups using an RNeasy kit (Qiagen) according to the manufacturer’s instructions, and RNA quality was assessed using a 2100 Bioanalyzer (Agilent). RNA with an RNA integrity number (RIN) > 8 was used for RNA sequencing with an Illumina TruSeq Stranded Total RNA Kit, which generated libraries following ribodepletion using a Ribo-zero Human/Mouse/Rat Kit. The Illumina HiSeq 2500 sequencing platform was used to sequence paired-end 100 bp runs (TheragenBio, South Korea).

RNA-seq preprocessing was performed with the automated IntelliseqFlow pipeline (https://intelliseq.com/). Within the pipeline, fastq files were checked for quality with the FastQC tool (version FastQC-v0.11.9) and mapped to the GRCm39 reference genome from Ensembl (version 104) using the STAR tool (version 2.7.3a). Gene expression levels were determined using the featureCounts tool (version 2.0.0). GTF files from the Ensembl database (version 104) were used as a reference. The raw gene counts were loaded into RStudio (version 4.1.2) and analysed with the EdgeR (version 3.38.4) library. The R code used for data analysis is available in the project’s GitHub repository (https://github.com/ippas/portsmouth-darek-mdxbrain). Genes were deemed statistically significant with EdgeR’s quasilikelihood F tests (QLF) tests for given contrasts. The following contrasts were considered in each experiment: (1) genotype effect in each age group; (2) age effect in each genotype; and (3) interaction between age and genotype factors. The full statistical results are available in the Supplementary material (Supplementary Tables 1 and 2). A false discovery rate (FDR) < 0.1 was considered to indicate statistically significant differential expression (DE). For the analysis of electron transport chain and mitochondrial genes, a preselected list of genes was plotted on the heatmap without any additional filtering.

### RNA splicing analysis

For differential splicing analysis, we used rMATS 4.1.2 (Wang et al. 2024) with default parameters, enabling variable read length and ignoring novel splice site predictions. Bam mode was used, and aligned bam files were passed from the STAR (version 2.7.3a) (Dobin et al. 2013) aligner following two-pass alignment with default parameters to the mm39 genome. To identify significant differential splicing events, a cut-off with an absolute inclusion level difference (ILD) greater than 0.05 and an FDR adjusted p value cut-off of less than 0.05 was used. To further reduce false positives, only events with a mean inclusion junction count (IJC) greater than 3 in either the wild-type or mutant condition were included. Enrichment analysis was performed on genes containing differential splicing events using clusterProfiler 3.0.4 (Wu et al. 2021), and the results were filtered using an adjusted p value less than 0.05.

### Genome-scale metabolic model

The metabolic alterations were analysed using a highly curated Genome-Scale Metabolic Model (GSMM) of *Mus musculus* (Wang et al. 2021) sample-specific transcriptomic data using our previous approach (Angione and Stegle 2018). First, the dead-end metabolites and reactions were removed because these do not carry any fluxes and are not part of metabolic pathways. Then, the reversible reactions were converted to irreversible reactions, as this reduced the complexity of the model while preserving biological relevance. By converting reversible reactions into irreversible reactions, the metabolic model assumes a fixed directionality for each reaction and reduces the number of parameters that need to be estimated in the optimization process. To further construct a model for the wild-type and mdx strains, the model was constrained with metabolite and enzymatic concentrations based on the literature (Tracey et al. 1996, 1995). In particular, the upper and lower bounds of the reactions associated with the metabolites and enzymes were constrained with the maximum values of the metabolite and enzymatic concentrations. The transcriptomic data were further used to constrain the model and generate sample-specific GSMMs. The idea is to constrain the upper and lower bounds of the metabolic models with sample-specific gene expression data, as illustrated in Eq. 1.

1 $$\begin{gathered} lb(i) = lb(i)*\left(geneReactionExpr(i)^{(gamma)} \right) \hfill \\ ub(i) = ub(i)*\left(geneReactionExpr(i)^{(gamma)} \right) \hfill \\ \end{gathered}$$

where *lb* is the lower bound and *ub* is the upper bound of the reaction, *i* is the index of the reaction, geneReactionExpr is the normalized expression value mapped to the reactions based on gene‒protein-reaction (GPR) rules for each sample and *gamma* is the hyperparameter used to constrain the model based on the gene expression value. Here, *gamma* was chosen to be 2. The sample-specific models were then solved using Flux Variability Analysis (FVA) and the ‘ATP maintenance requirement’ reaction as objective functions to evaluate the minimum and maximum flux rates for each reaction, satisfying the given constraints.

Similar to our previous experiment (Gosselin et al. 2022), the flux fold changes of mdx and the wild type were estimated as described in Eq. 2.

2 $$fluxfoldchange = \frac{{MaxFlux_{mdx} + 1}}{{MaxFlux_{wildtype} + 1}},$$

where $MaxFlu{x}_{mdx}$ is the Max Flux from the mdx-specific GSMM and $MaxFlu{x}_{wildtype}$ is the Max Flux from the wild-type GSMM. To avoid 0/0 or NANs, a correction of +1 was added to both the mdx and wild-type max fluxes.

The enrichment analysis of reactions and pathways was carried out by identifying the upregulated and downregulated reactions and pathways by using a threshold value for log2-fold change required to obtain the expected enriched reactions and pathways. The reactions with a log2 (flux fold change) greater than 1.5 and a log2 (flux fold change) distribution above the 95th percentile were classified as “upregulated reactions”, while the reactions with a log2 (flux fold change) less than −1.15 and below the 5th percentile log2 (flux fold change) were classified as “downregulated reactions”.

Similarly, the upregulated and downregulated pathways were also estimated by grouping the fold change of reactions by pathways.

Specifically, since multiple reactions can be associated with the same pathway, the fold change in each metabolic pathway between the 10d mdx and wild-type strains and between the 10w mdx and wild-type strains was calculated by averaging the flux fold changes of the reactions belonging to that specific pathway. Pathways with a log2 (flux fold change) greater than 1 and a log2 (flux fold change) distribution above the 95th percentile were classified as "upregulated pathways", while pathways with a log2 (flux fold change) less than 0 and below the 5th percentile log2 (flux fold change) were classified as “downregulated pathways”.

### Quantitative PCR

Quantitative PCR (qPCR) was conducted using specific primer pairs (Table 1) designed using NCBI-primer BLAST or previously published primers (Crawford et al. 2023; Kawalec et al. 2015; Masin et al. 2012). Total RNA was extracted as previously described and converted to cDNA using the SuperScript VILO cDNA Synthesis Kit (Invitrogen). qPCRs were performed using PowerTrack SYBR Green master mix (Applied Biosystems) on a QuantStudio 5 real-time PCR system (Applied Biosystems) according to the manufacturer’s instructions. The results were normalized to the expression levels of housekeeping genes (ubiquitin C (*Ubc)* and zinc finger protein 91 (*Zfp91)*), which were previously identified as being stably expressed in wild-type and dystrophic cerebra and cerebella (Crawford et al. 2023), using the 2-ΔΔ Ct method. The mtDNA content was measured as described in Kawalec et al. (2015) using primers for the mouse mitochondrial displacement loop (D-loop) region (mtDNA) and single-copy nuclear thymidylate kinase gene (nuclear DNA). The primers used are shown in Table 1.

**Table 1:** List of primers used in this study

Primers used		
Ubc	Forward	5′- GCCCAGTGTTACCACCAAGA-3′
	Reverse	5′-CCCCATCACACCCAAGAACA-3′
Zfp91	Forward	5′-CCCGGTGGCATTAGTAGTGA-3′
	Reverse	5′-CTGATTTTCTCCGTGGCTTTGG-3′
Dp427m	Forward	5′-GGGAAGAAGTAGAGGACTGTTATG-3′
	Reverse	5′-GGTTGTCTATGTGTTGCTTTCC -3′
Dp427p	Forward	5′-TTTGTCAGGCTGCGTAGAGA-3′
	Reverse	5′-AGGACAAACCTGGAGGTAGAGT-3′
Dp427c	Forward	5′-CAGGAGAAAGATGCTGTTTTGC-3′
	Reverse	5′-TTCCTGTCACTCCATCATGCC-3′
Dp71	Forward	5′-TTGGGCAAGCTTACTCCTCC-3′
	Reverse	5′-TTTGGGTCTCGTGGCCTTT-3′
Dp140	Forward	5′-TCTGAGCTAAAATCGTCAGTGT-3′
	Reverse	5′-AATGCCATCCTGGAGTTCCTTAAT-3′
P2RX7a NT	Forward	5′-GCACGAATTATGGCACCGTC-3′
	Reverse	5′-TAACAGGCTCTTTCCGCTGG-3′
P2RX7k	Forward	5′-TATGGATCGGGATGAAG-3′
	Reverse	5′-GTGTGCACGGAGCTGATAAC-3′
P2RX7 (Exon 9)	Forward	5′-GAGAACAATGTGGAAAAGCGG-3′
P2RX7a CT	Reverse	5′-TCAGTAGGGATACTTGAAGCC-3′
P2RX7b	Reverse	5′-TCAGGTGCGCATACATACATG-3′
P2RX7c	Reverse	5′-TCTGTGAGAAACAAGTATCTAGGTTGG-3′
mtDNA	Forward	5′-CCAAAAAACACTAAGAACTTGAAAGACA-3′
	Reverse	5′-GTCA TATTTTGGGAACTACTAGAATTGATC-3′
Nuclear DNA	Forward	5′-GACTGTATTGAGCGGCTTCAGA-3′
	Reverse	5′-CATGCTCGGTGTGAGCCATA-3′

The *P2RX7 (Exon 9), P2RX7a CT, P2RX7b, P2RX7c* (Masin et al. 2012), mouse mitochondrial displacement loop (D-loop) and thymidylate kinase genes were used as mitochondrial and nuclear DNA, respectively (Kawalec et al. 2015). *CT* C-terminal, *NT* N-terminal

### Antibodies

The following antibodies were used for Western blotting: anti-P2X7 (177003, Synaptic Systems), anti-P2X4 (APR-002, Alomone Labs), and anti-GLUT3+14 (ab191071, Abcam) at a 1:1000 dilution. Anti-GLUT1 (ab191071, Abcam) was used at a dilution of 1:10,000. The OxPhos Rodent WB Antibody Cocktail (Thermo Fisher Scientific), consisting of primary antibodies recognizing V-ATP5A (55 kDa), III-UQCRC2 (48 kDa), IV-MTCO1 (40 kDa), II-SDHB (30 kDa), and I-NDUFB8 (20 kDa), was also used according to the manufacturer’s instructions with a 1:1000 dilution. For immunohistochemistry, rabbit anti-GABA(A) α1 receptor (AGA-001, Alomone labs) at a 1:2000 dilution, rabbit anti-mouse P2Y12R (AnaSpec Inc., Cat# As-55043a), chicken anti-Iba1/AIF1 (Alves Labs, IBA1-0100), and guinea pig anti-mouse CD39 (mN1-2cI5, http://ectonucleotidases-ab.com/ [gift of Prof. Jean Sevigny, Quebec, Canada]) antibodies were used (1:800). Alexa Fluor® 488-conjugated anti-chicken (A11039) and Alexa Fluor® 633-conjugated anti-rabbit (AA21071) secondary goat antibodies were obtained from Invitrogen (Thermo Fisher Scientific), and Alexa Fluor® 488-conjugated anti-rabbit (711-545-152) and Cy3™-conjugated anti-guinea pig (706-165-148) donkey antibodies were obtained from Jackson ImmunoResearch Laboratories.

### Western blotting

Total proteins were extracted from tissue powders obtained by crushing samples under liquid nitrogen with further homogenization in ice-cold extraction buffer, which was either RIPA buffer (150 mM NaCl, 1% Triton X-100, 0.5% sodium deoxycholate, 0.1% sodium dodecyl sulfate, and 50 mM Tris, pH 8.8) containing 10 mM sodium fluoride, 2 mM sodium orthovanadate and an EDTA-free protease inhibitor cocktail tablet (Roche) or cell lysis buffer (Cell Signaling Technology) supplemented with 1 mM PMSF (Bio-Rad). For RIPA buffer extraction, the samples were left on a roller at 4 °C for 2 h and centrifuged at 14,000×*g* for 20 min. For the cell lysis buffer method, lysates were left on ice for 5 min, sonicated 4 times for 5 s (Vibra-Cell™ SYSTEM 130, 130 W, with 38 mm Micro Cup Horn; SONICS & MATERIALS) and centrifuged at 14,000×*g* for 10 min at 4 °C. The protein concentration was determined using a bicinchoninic acid (BCA) assay (Thermo Fisher Scientific) or a modified Lowry protein assay (Thermo Fisher Scientific). Protein (10–30 µg) was mixed with 2× SDS Tris glycine sample buffer (Novex, Invitrogen) at a 1:1 v/v ratio with 2.5% v/v β-mercaptoethanol. Samples were heated to 90 °C for 10 min, except when anti-GLUT1 (ab191071, Abcam) and OxPhos Rodent WB Antibody Cocktail (Thermo Fisher Scientific) were used. The samples were separated on SDS–polyacrylamide gels with varying acrylamide concentrations (10% or 15%) or on Any kD Mini-PROTEAN TGX Precast Protein Gels (Bio-Rad), depending on the size of the proteins to be detected. Following electrophoresis, proteins were transferred onto 0.45 µm Protran Supported Nitrocellulose Blotting Membranes (Amersham or Whatman GmbH). Protein transfer was assessed by reversibly staining the membrane with 0.1% Ponceau S and/or staining the posttransfer gel with Coomassie Brilliant Blue (CBB) (NIH, Bethesda, MD, USA). The membranes were blocked in 5% nonfat milk powder in 1× Tris-buffered saline with 0.01% Tween 20 (TBST) for one hour prior to being probed with a primary antibody overnight at 4 °C using the antibody dilutions described above. The membranes were washed 4× for 10 min with TBST and incubated with the appropriate horseradish peroxidase-conjugated secondary antibodies: anti-rabbit (1:5000) (Sigma Aldrich, A6154) and anti-mouse (Sigma‒Aldrich, A9044). The membranes were then washed 3 × 5 min. The western blot signal was developed with ECL Western Blotting Detection Reagent (Amersham) or Immobilon Crescendo HRP Substrate (Merck) and then detected and quantified using a Fusion FX imaging system (Vilber Lourmat, Marne-la-Vallée, France) or a ChemiDoc MP system (Bio-Rad). Densitometric analyses were performed using the integrated density measurement function of ImageJ software, and the proteins were normalized to the total protein visualized by Ponceau S or CBB. All experiments were repeated at least three times.

### Immunolocalization

Animals were anaesthetized, transcardially perfused with a 0.9% saline solution for 2 min and perfusion-fixed for 10 min with 4% paraformaldehyde in 0.1 M phosphate buffer (PB), pH 7.4. The brains were dissected and postfixed overnight at room temperature (RT). The same fixative was used, followed by a saturated sucrose solution. Frontal sections (30 μm) were prepared using a HYRAX M25 rotary microtome (Zeiss, Germany) equipped with an MTR quick-freezing unit (SLEE, Germany). Each section was stored in PBS supplemented with 0.01% w/v sodium azide in a 96-well plate at 4 °C until further processing. The free-floating staining method was adapted from Tu et al. (2021). For GABA_A_R α1 detection, cerebellar sections were permeabilized in PBS with 0.25% Triton X-100 for 10 min and blocked in a solution containing PBS, 0.1% Triton X-100 and 2% BSA for 1 h at RT. For sections that were not permeabilized, Triton X-100 was omitted from every staining solution. Next, the sections were incubated for 24 h at RT with an anti-GABA(A) α1 receptor antibody (1:2000) in blocking solution, washed and incubated for 2 h at RT with Alexa Fluor 488-conjugated donkey anti-rabbit IgG (1:1000). The nuclei were stained with Hoechst 33342 (1:10,000, Invitrogen). The sections were then mounted with ProLong Glass Antifade Mountant (Thermo Fisher Scientific) and imaged using a Zeiss LSM780 Axio Observer confocal microscope (Carl Zeiss AG, Oberkochen, Germany). Images were acquired using a 63× Plan-Apochromat oil immersion objective (1.4 NA). The unidirectional scanning mode was used, and the image resolution was 1024 × 1024 pixels. The laser power, detector gain, frame time and histogram values were set and reused throughout the image acquisitions. Fluorescence intensity measurements were taken using ZEN 2.6 (blue edition) software (Carl Zeiss) from the Purkinje cell area outlined only with the Draw Spline Contour Tool, and the arithmetic mean of the fluorescence intensity was read for every image.

For microglial analysis, brains were fixed for 2 h at RT in 4% paraformaldehyde (PFA) in PBS and embedded in a mold with 4% NuSieve™ GTG™ low melting agarose (LMA) (Lonza) solution in PBS. LMA-embedded brains were sectioned at 100 µm using a Leica VT1200S vibrating microtome and additionally fixed for 30 min with 4% PFA. Next, the sections were incubated for 1 h at RT in 300 μl of blocking buffer containing 2% bovine serum albumin (BSA) and 0.5% (vol/vol) Triton X-100 in PBS and subsequently incubated with primary antibodies diluted in 300 μl of the same blocking buffer overnight at 4 °C. The samples were incubated overnight at 4 °C with the appropriate fluorochrome-conjugated secondary antibodies diluted in blocking buffer at ~1:800. All staining procedures were performed in a 24-well plate under 60 rpm orbital rotation, with 3 × 30 min washes between each treatment with 300 μl of blocking buffer. Finally, the stained sections were washed for 10 min in 500 μl of PBS and mounted with ProLong® Gold Antifade reagent containing 4,6-diamidino-2-phenylindole (DAPI) (Thermo Fisher Scientific), with glass spacers inserted between the slide and the coverslip. Imaging was performed using a 3i CSU-W1 spinning disk confocal microscope (Intelligent Imaging Innovations, Inc.) equipped with a Hamamatsu ORCA Flash 4 sCMOS camera (Hamamatsu Photonics, Hamamatsu, Japan) and Slidebook 6.0 software. Z-stacks of single images of the frontal lobe and cerebellum were captured using an LD C-Apochromat 40×/1.1 objective. Maximum intensity projections and 3D reconstructed images were prepared using Imaris 8.4 software (Bitplane).

### In situ enzyme histochemistry

For enzyme histochemistry, the brains were mounted on cork disks with Neg-50^TM^ OCT-embedding matrix (Epredia, Fisher Scientific) and frozen in isopentane (Fisher Scientific) that had been chilled in liquid nitrogen. 10 μm sections were cut onto polylysine^TM^ adhesion microscope slides (Epredia, Fisher Scientific) using a Leica CM 3050S cryostat, air-dried and stored at −80 °C. For localization of ectonucleotidase and tissue-nonspecific alkaline phosphatase (TNAP) activities, a histochemical approach was employed (Losenkova et al. 2020). In brief, brain cryosections were thawed, fixed for 5 min with 4% PFA, and preincubated for 45 min at RT in Tris-maleate sucrose buffer (TMSB) [40 mmol/L Trizma-maleate, 0.25 mol/L sucrose, pH 7.4] supplemented with the TNAP inhibitor tetramisole (2 mM) (Sigma Aldrich). The slides were subsequently incubated for one hour at RT in a mixture containing TMSB (pH 7.4), 2 mM tetramisole, 2 mM Pb(NO_3_)_2_, 0.5 mM CaCl_2_ and 300 μM ATP serving as the preferred substrate for CD39. The lead orthophosphate precipitated in the course of the ATPase reaction was visualized as a brown deposit by incubating the sections for 15 s in 0.5% (NH_4_)_2_S. TNAP activity was evaluated by measuring the intensity of the dark purple precipitate after incubating the tissues for 20 min at RT in a mixture containing TMSB (pH 9.3), 5 mM MgSO_4_ and the artificial enzyme substrates 5′-bromo-4-chloro-3-indolylphosphate (BCIP) and nitro blue tetrazolium (NBT) (0.2 mmol/L) (Sigma Aldrich). Whole-tissue section imaging was performed using a Pannoramic P1000 slide scanner (3DHistech Ltd., Budapest, Hungary) with a 20× objective.

### Mitochondrial isolation

The mitochondria-enriched fraction was isolated as previously described (Andersen et al. 2017; Ju et al. 2020; Yonutas et al. 2020). The freshly dissected tissue was homogenized in mitochondrial isolation buffer (210 mM mannitol, 70 mM sucrose, 5 mM HEPES, 1 mM EGTA and 0.5% (w/v) fatty acid–free BSA, pH 7.2) using a Teflon-glass homogenizer. The homogenates were centrifuged at 1000×*g* for 10 min at 4 °C, and the resulting supernatants were centrifuged at 11,000×*g* for 20 min at 4 °C. The crude mitochondrial pellets were resuspended in ice-cold BSA-free mitochondrial isolation buffer. The protein concentration of the mitochondrial samples was determined using a Bradford protein assay (Bio-Rad). The mitochondrial suspensions were kept on ice and used immediately for the Seahorse assay.

### Seahorse assay

The oxygen consumption rate of the extracted mitochondria was measured using an XFe96 Seahorse analyser (Agilent). The XFe96 sensor cartridge was hydrated overnight according to the manufacturer’s instructions. The XFe assay compounds were diluted in mitochondrial assay solution (MAS: 220 mM mannitol, 70 mM sucrose, 10 mM KH_2_PO_4_, 5 mM MgCl_2_, 2 mM HEPES, pH 7.2, 1 mM EGTA, 0.2% BSA) supplemented with respiratory substrates, 1 mM pyruvate, 5 mM malate, 10 mM glutamate, 5 mM ADP with or without 10 mM succinate, and oligomycin (5 μM), rotenone (2 μM), digitonin (1 µg/mL), FCCP (5 μM), and antimycin A (10 μM) were added. Mitochondria (3 µg/50 µL per well) diluted in MAS were added to the appropriate wells; pure MAS containing respiratory substrates was also added to the reference wells. The assay plate was centrifuged at 2000×*g* for 20 min at 4 °C. MAS buffer with substrates prewarmed to 37 °C (130 µL) was added to each well. The assay plate was incubated for 10 min at 37 °C prior to measurement. The standard protocol for the isolation of mitochondria was followed, except that the order of the drugs used was oligomycin, rotenone, FCCP, and antimycin A. Two time points were measured for each step of the experiment. The data were analysed using Agilent Seahorse Analytics Online Analysis Software and GraphPad Prism 9.

### Statistical analysis

Statistical analysis was performed with GraphPad Prism 9. The Shapiro‒Wilks test was first used to assess whether the data were normally distributed. If the data were normally distributed, comparisons between two groups were made using Student’s unpaired t test, while multiple group comparisons were performed using two-way analysis of variance (ANOVA) with Tukey’s test to correct for multiple comparisons in the post hoc analysis. If the data were not normally distributed, the Mann‒Whitney U test was used for comparing two groups, and the Kruskal‒Wallis test was used for multiple group comparisons. In the latter case, Dunn’s test was applied to correct for multiple comparisons. A *p* value < 0.05 was considered to indicate statistical significance, and the *p* values are reported as follows: *p < 0.05, **p < 0.005, ***p < 0.001.

## Results

Total RNA was extracted from the cerebra and cerebella of 10-day-old (10d) and 10-week-old (10w) mdx and C57Bl/10 mice subjected to RNA-Seq, and the differentially expressed genes (DEGs) were investigated. This comparison identified specific pathways enriched in DEGs that occur in these two brain regions due to the absence of full-length dystrophin before (10d) and after (10w) the onset of chronic muscle inflammation.

### Dystrophin transcript expression in the cerebra and cerebella

**Fig. 1:**
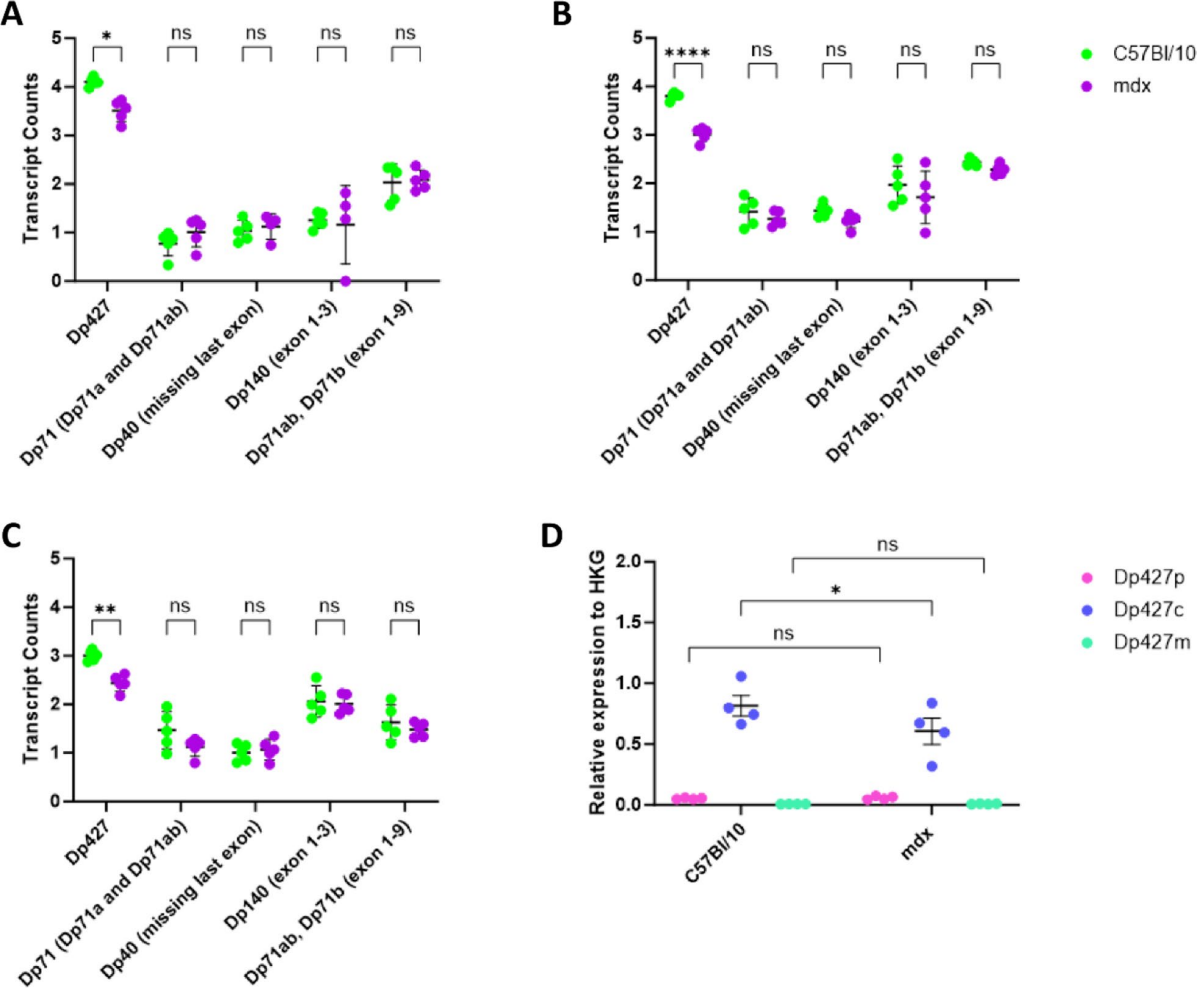
Expression of dystrophin transcripts in the cerebra and cerebella. **A** RNA-Seq dystrophin transcript expression data in 10w mdx and C57Bl/10 cerebra **B** in 10d mdx and C57Bl/10 cerebra and **C** in 10w mdx and C57Bl/10 cerebella. **D** qPCR analysis of the expression levels of specific full-length dystrophin transcripts (Dp427c, Dp427p, Dp427m) in 10w mdx and C57Bl/10 cerebra. *HKG* housekeeping gene. The significance was analysed using the Shapiro‒Wilk test to confirm normality and 2-way ANOVA with Tukey’s post hoc test; *p value < 0.05, **p value < 0.01, ****p value < 0.0001, *ns* not significant

To investigate the potential upregulation of shorter dystrophins that might occur in the cerebra and cerebella of mdx mice, RNA-seq data and qPCR analysis were used to study the expression of mRNAs encoding specific dystrophin isoforms. This analysis confirmed the predominant expression of *Dp427c* in the wild-type cerebrum and no significant expression of the *Dp427m* variant in these brain regions. RNA-Seq confirmed the expected decrease in the expression of full-length dystrophin transcripts in the cerebra of 10w and 10d mdx mice and in the cerebella of 10w mdx mice (Fig. 1a–c), but no significant difference was found in the cerebella of 10d mice (Supplementary Fig. 2a), which coincided with very low expression levels at this age. qPCR analysis of specific transcripts revealed that cerebellar expression of *Dp427c* at both time points was significantly downregulated in adults (Supplementary Fig. 2b, c). *Dp427c* expression was reduced in the cerebra of 10w mdx mice (*p* = 0.0169) but not in the cerebra of 10d mdx mice (Fig. 1d, Supplementary Fig. 2d). No significant difference in transcripts encoding the *Dp71* and *Dp140* shorter dystrophin isoforms found at 10d and 10w ruled out compensatory overexpression influencing the dystrophic cerebral or cerebellar phenotypes (Fig. 1a–c,Supplementary Fig. 3).

### Transcriptomic alterations in dystrophic cerebra are substantial and affect basic cellular processes at both 10d and 10w

**Fig. 2:**
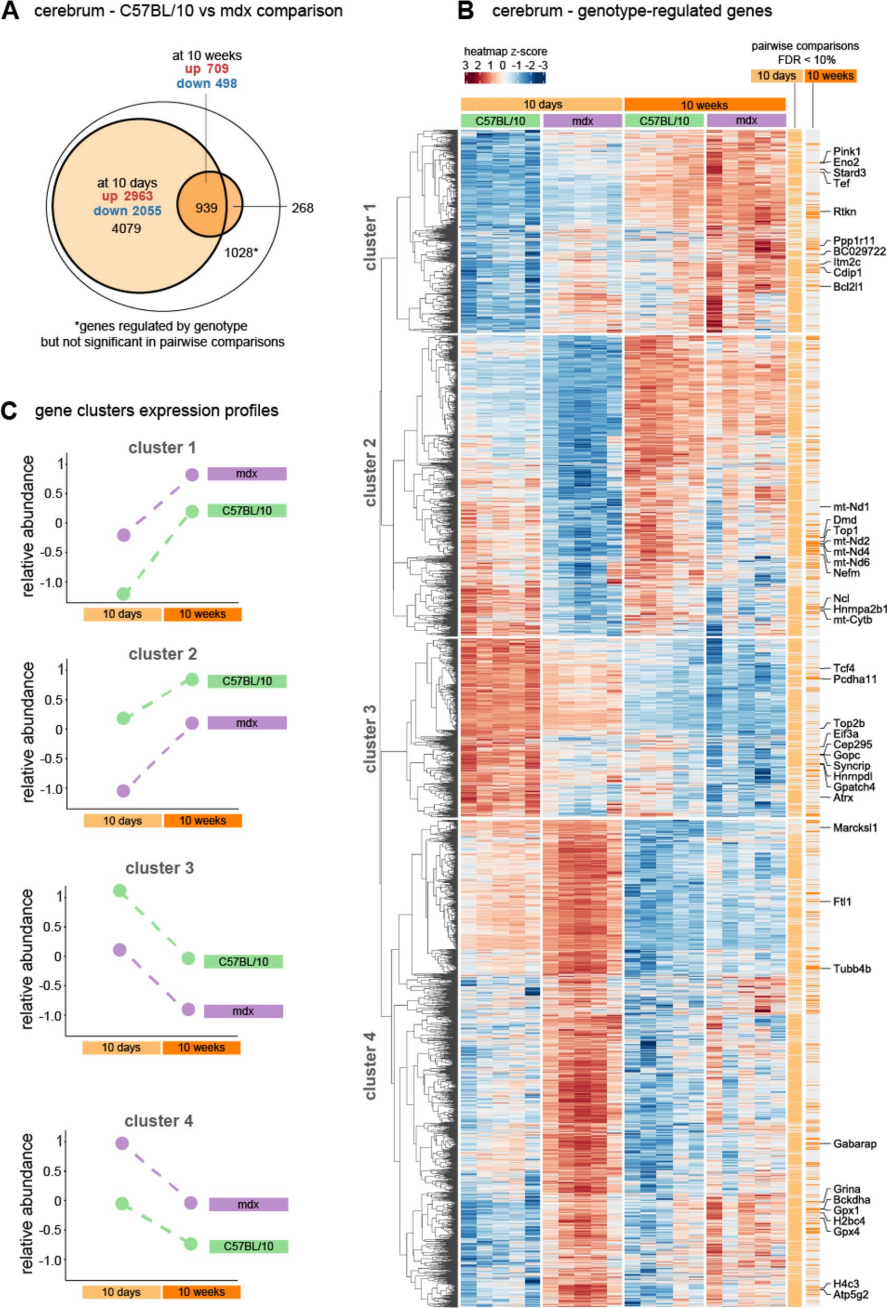
Differential gene expression in 10w and 10d mdx and C57Bl/10 cerebra. **A** Venn diagram showing the number of genes differentially expressed (FDR 10%) between mdx and C57BL/10 mice. The *largest ellipse* contains all of the genes regulated by genotype (both ages considered together). The *smaller circles* show genes that were also significantly altered in pairwise comparisons between mdx and C57BL/10 mice at each age. **B** Heatmap showing all genes regulated at an FDR < 10% according to the genotype factor. The top 10 genes within each cluster and the lowest genotype FDR are marked for clarity. The intensity of the *coloured rectangles* represents transcript abundance levels. The presented level is proportional to the row z score values (between *darkest blue*: −3 and *darkest red*: 3), as displayed on the bar above the heatmap image. Hierarchical clustering was performed using correlation as a distance measure. The *light* and *dark orange bars* highlight whether the gene is differentially expressed (FDR < 10%) in 10d and 10w mdx cerebra compared to those in the cerebra of age-matched controls. A full list is available in Supplementary Table 1. **C** Profiles of differences for the four identified clusters of altered genes; mean transcript abundance levels are presented for each group of genes

In the cerebra, we identified four major clusters of differentially expressed genes (DEGs) between genotypes (overall mdx vs C57BL/10 effect). Altogether, the clusters contained 6314 DEGs at the assumed False Discovery Rate (FDR) threshold of 10% (Fig. 2a, b). The threshold was chosen for the consistency of transcriptomic analyses performed throughout the study. According to pairwise comparisons, 77.0% of all the DEGs were altered in 10d animals, while 19.9% were altered in older animals, indicating that the mutation effect is more pronounced earlier in development.

Moreover, although more than 10 000 cerebral transcripts were altered by age, no interaction between genotype and age was detected (FDR 10%, full results available in Supplementary Table 1). This suggests that the genotype-induced transcriptomic changes should be largely similar in both age groups. Indeed, a detailed analysis of the expression profiles of each of the clusters indicated that, in the cerebra, the mdx mutation tended to affect genes in the same direction regardless of the age group, although the differences were smaller in the 10w animals (Fig. 2c). The four clusters divided DEGs into upregulated (1 and 4) or downregulated (2 and 3) DEGs according to genotype and then into upregulated (1 and 2) and downregulated (3 and 4) DEGs according to age.

**Fig. 3:**
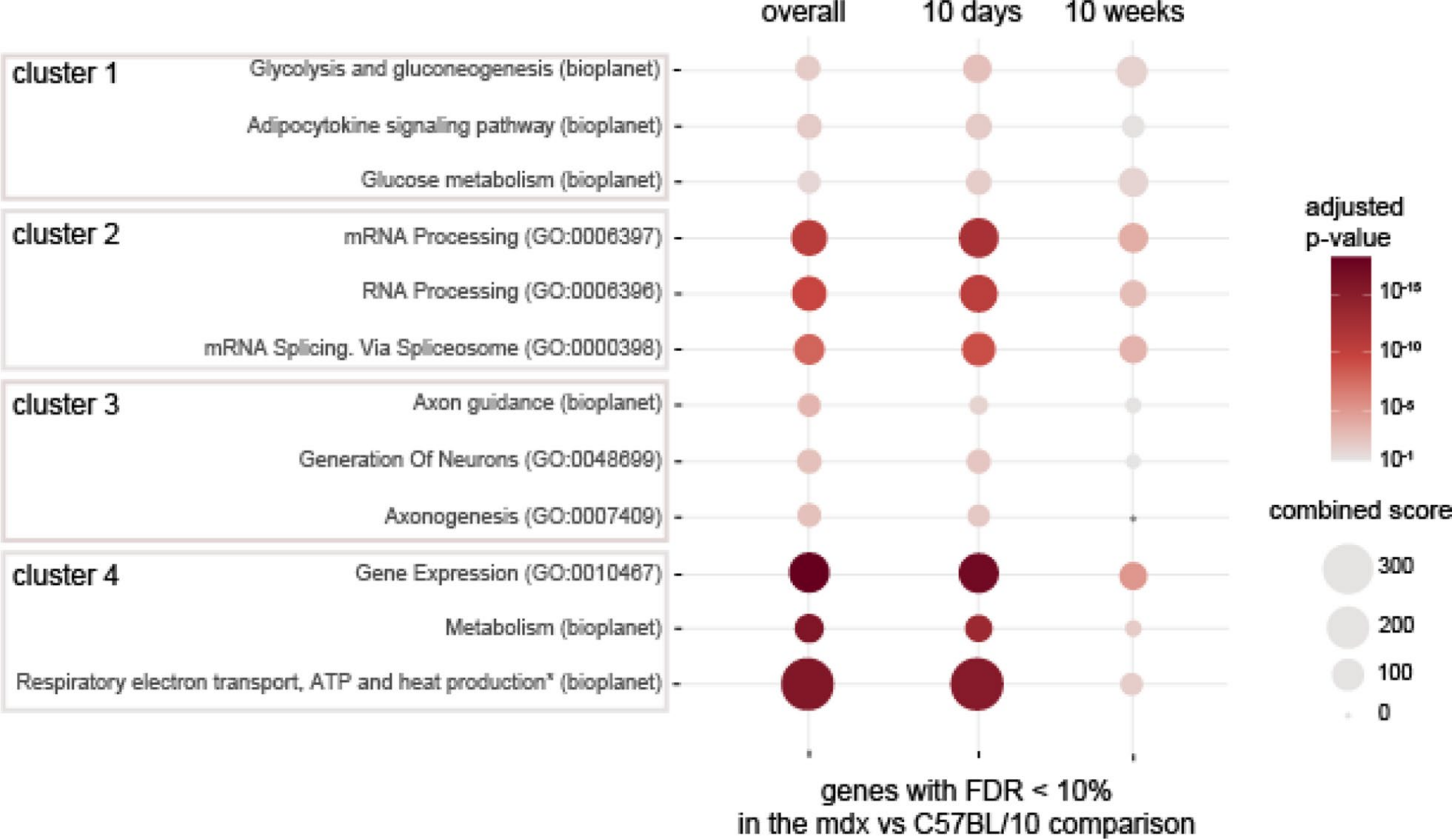
Biological processes and molecular pathways associated with genes differentially expressed in the cerebra of mdx mice. For each of the clusters, the top three unique enriched terms (ranked by adjusted p value) from the Bioplanet and GO Biological Process databases were selected. The full enrichment results are available in Supplementary Tables 3 and 4. The size of each circle reflects Enrichr’s combined score (calculated from the *p* value and odds ratio). The colour reflects the *p* value (the darker the colour is, the lower the *p* value). The first column shows the results from the full gene list from each cluster, while the second and third columns display the results for the same terms for the gene lists filtered to only contain genes significant in pairwise comparisons in each of the ages. * term description was shortened, full name: Respiratory electron transport, ATP biosynthesis by chemical coupling, and heat production by uncoupling proteins

For each of the clusters, we performed gene enrichment analysis with EnrichR, which allows for the interrogation of multiple databases in one analysis (Chen et al. 2013; Kuleshov et al. 2016; Xie et al. 2021). The results from two databases, (1) GO Biological Process, which describes biological modules or programmes, and (2) NCATS Bioplanet, which curates molecular pathways, were selected as the most informative (Supplementary Tables 3, 4). For each cluster, pathway enrichment was analysed for (1) overall genotype effect, (2) genes affected by genotype in 10d animals, and (3) genes affected by genotype in 10w animals. The top 3 unique terms according to the adjusted p value rankings are displayed in Fig. 3. For clusters with genes upregulated in mdx mice (clusters 1 and 4), the top enriched terms were associated with carbohydrate metabolism (cluster 1) and basic cellular metabolism, including respiration (cluster 4). Genes downregulated in mdx animals (clusters 2 and 3) were associated with mRNA processing (cluster 2) and neuronal development (cluster 3). For all of the gene lists, the enrichment was more pronounced (as defined by the adjusted p value) in the 10d cerebra. However, many terms were still significantly enriched in 10w animals (all of the top terms for clusters 2 and 4). Alterations in inflammatory pathways and relevant DEGs are discussed separately below.

### Full-length dystrophin loss and cerebral glucose metabolism

**Fig. 4:**
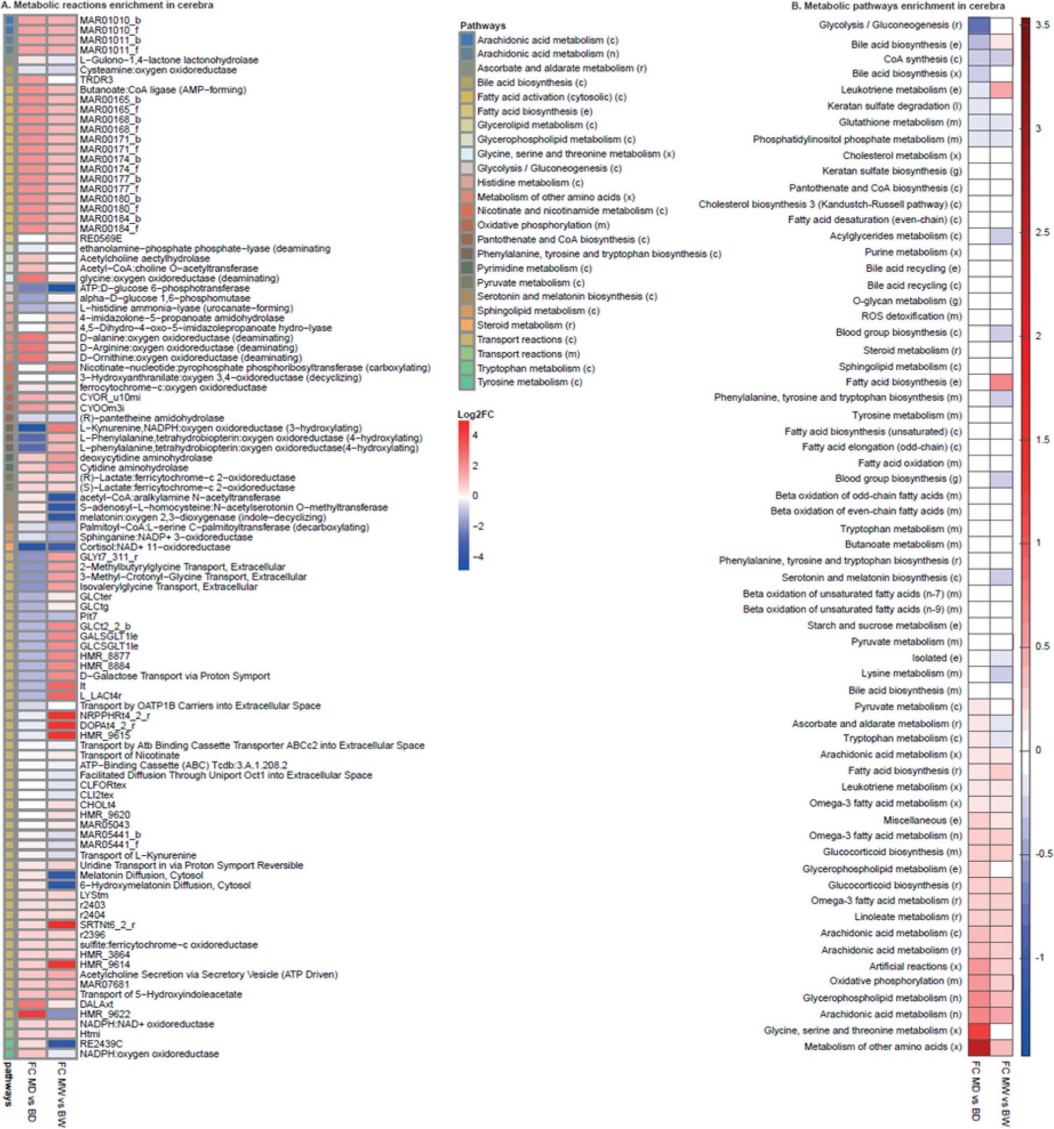
Dystrophic genome-scale metabolic modelling. **A** Reaction enrichment analysis. Fold changes in the reaction flux between mdx and C57Bl/10 at 10d and 10w. The reactions are sorted by pathway, and the pathways associated with each reaction are indicated. **B** Fold changes in pathway flux between the cerebra of mdx and C57Bl/10 mice at 10d and 10w. The reactions are grouped by pathways, indicating the pathways associated with each reaction. For **A** and **B**, the upregulated and downregulated reactions are shown in *red* and *blue*, respectively; “_b” and “_f” represent backwards and forward reactions, respectively; compartments: (e): Extracellular, (x): Peroxisome, (m): Mitochondria, (c): Cytosol, (l): Lysosome, (r): Endoplasmic reticulum, (g): Golgi apparatus, (n): Nucleus, (i): Inner mitochondria

Given the transcriptomic findings that energy metabolism is known to be altered in the mdx mouse brain (Rae et al. 2002; Tracey et al. 1996; Tuon et al. 2010) and that inflamed tissues show significant changes in metabolic activity (Kominsky et al. 2010), we investigated the metabolic landscape. A dystrophic genome-scale metabolic model (GSMM) was reconstructed following our established pipelines for generating context-specific metabolic models (Fig. 4).

**Fig. 5:**
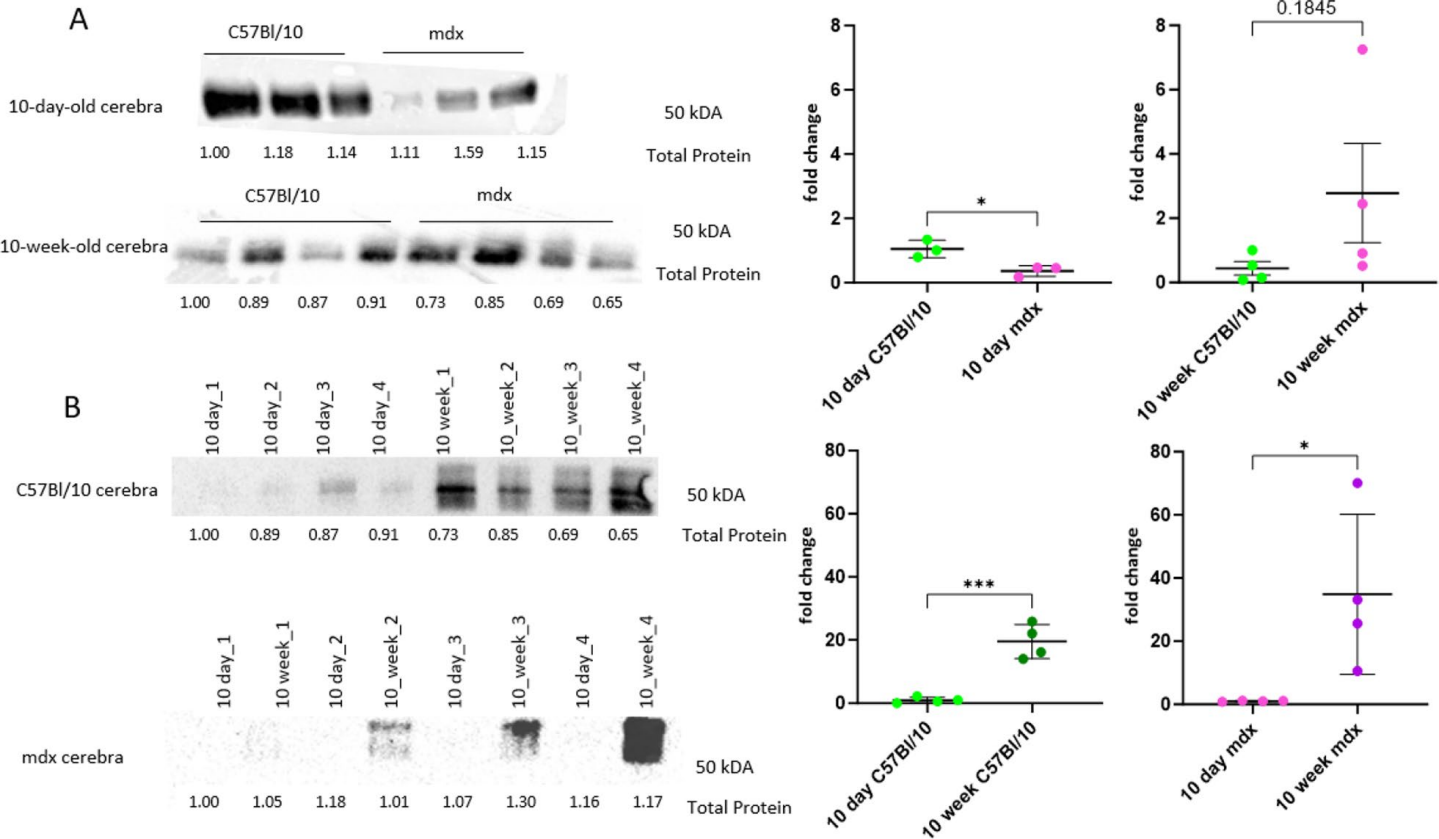
Expression of GLUT1 in mdx and C57Bl/10 cerebra **A** GLUT1 in 10d mdx and C57Bl/10 cerebra and 10w mdx and C57Bl/10 cerebra. **B** GLUT1 expression in the cerebra of 10d and 10w C57BL/10 mice and mdx mice. GLUT1 was detected by western blot and normalized to total protein in lines visualized with Ponceau S. Normalization factors are shown below the representative western blot images. The graph shows the mean values with SDs. Statistical significance was calculated using Student’s *t* test; n = 3; *p < 0.05, ***p < 0.001. The blot represents one of at least three repeats

Specific upregulated and downregulated reactions and pathways for 10d and 10w dystrophic cerebra compared to age-matched controls were identified (Fig. 4a, b). In young cerebra, the reactions in the glycolysis/gluconeogenesis pathway were found downregulated, suggesting a significant impact on cellular energy metabolism. Specifically, the ‘ATP: D-glucose 6-phosphotransferase’ and ‘alpha-D-glucose 1,6-phosphomutase’ reactions in this pathway were downregulated at 10d. However, in 10w dystrophic cerebra, the ‘alpha-D-glucose 1,6-phosphomutase’ reaction was upregulated. The reduced activity in the glycolysis/gluconeogenesis pathway indicates a decrease in glucose metabolism and suggests that mitochondrial metabolism could be a compensatory mechanism to maintain energy production. The mitochondrial oxidative phosphorylation pathway was upregulated, especially the respiratory complex IV Cytochrome c oxidase subunit III (CYOOm3i) and ferrocytochrome-c: oxygen oxidoreductase reactions were upregulated at 10d and 10w, suggesting increased mitochondrial metabolism. Then, the reactions linked to glucose transport were analysed, and GLCter and GLCtg, which are involved in the transport pathway, and GLCt2_2, GLCSGLT1le and GLCt4_2, which are responsible for glucose transport, were found to be downregulated in the young cerebra but upregulated in the mature cerebra. Thus, metabolic modelling revealed significant dysregulation of glucose metabolism in the glycolysis/gluconeogenesis and transport reaction pathways between 10d and 10w cerebra. Interestingly, while according to the transcriptomic data, the expression of the *Slc2a1* gene in 10d dystrophic cerebra was significantly increased, the expression of the glucose transporter 1 (GLUT1) protein encoded by this gene was significantly downregulated at this age (Fig. 5a). At 10w, GLUT1 expression levels showed high intragroup variability, and there was no statistically significant difference between the genotypes (Fig. 5a). Interestingly, the expression of this protein increased significantly between 10d and 10w in both dystrophic and C57Bl/10 mice, with a much greater fold change in the cerebra of mdx mice (Fig. 5b). GLUT3+14 was also upregulated at the transcriptomic level, but there was no significant difference at the protein level in the mdx cerebra (Supplementary Fig. 4).

### Mitochondrial alterations in dystrophic cerebra are age-dependent

**Fig. 6:**
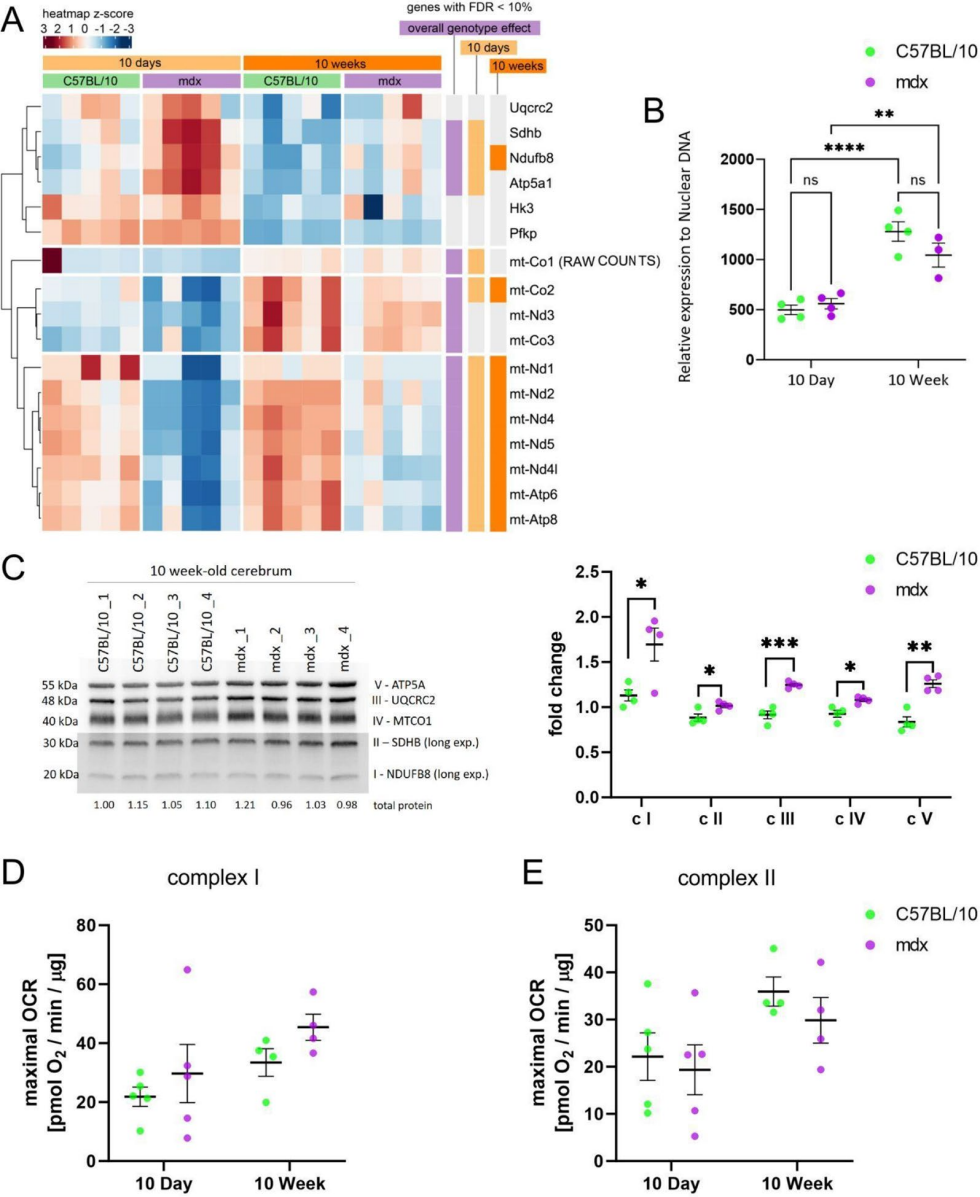
Mitochondrial alterations in 10d and 10w mdx cerebra due to full-length dystrophin loss. **A** Heatmap of the abundance of selected ETC genes in the cerebra. The intensity of the *coloured rectangles* represents transcript abundance levels. The presented level is proportional to the row z score values (between *darkest blue*: −3 and *darkest red*: 3), as displayed on the bar above the heatmap image. To order rows (genes), hierarchical clustering was performed using correlation as a distance measure. The annotations in columns on the right of the heatmap highlight whether the gene is differentially expressed (FDR < 10%) according to the global genotype effect and then in 10d and 10w mdx cerebra compared to age-matched controls. RAW COUNTS: mt-Co1 is displayed as raw data (without normalization), as it was the most abundant transcript in all of the samples; therefore, quantile-normalized counts (as for other genes) are not suitable for visualization. All of the genes apart from Uqcrc2 and mt-Nd4l were also differentially expressed between ages (FDR 10%). **B** Mitochondrial DNA content is presented as the mitochondrial to nuclear DNA ratio (mtDNA/nDNA); n = 5; **p < 0.01, ***p < 0.001. **C** Protein content of ETC subunits in 10w cerebra from C57Bl/10 and mdx mice. The ETC subunits NDUFB8 (complex I; c I), SDHB (complex II; c II), UQCRC2 (complex III; c III), MTCO1 (complex IV; c IV) and ATP5A (complex V; c V) were detected by western blotting and normalized to total protein in lines visualized with Ponceau S. Normalization factors are shown under the representative western blot images. The charts present the mean values with standard deviations. Statistical significance was determined with Student’s *t* test; n = 4; *p < 0.05, **p < 0.01, ***p < 0.001. The maximal oxygen consumption rate (OCR) of mitochondria isolated from 10d and 10w cerebra from C57Bl/10 and mdx mice, measured after the administration of FCCP for complex I (in the absence of the complex II substrate succinate and complex I inhibitor rotenone (**D**) and for complex II (in the presence of succinate and rotenone). **E** Two-way ANOVA was performed together with multiple comparisons *t* tests with Bonferroni correction. The charts present the mean values ± SDs. The number of animals included in the analysis was n = 5 for 10d and n = 4 for 10w animals

The transcriptomic analysis and metabolic GSMM modelling suggested potential alterations in mitochondrial function in the dystrophic cerebra. Specifically, analysis of the transcriptomic data revealed significant changes in the cerebral expression of glycolysis- and mitochondria-related genes (Fig. 6a), both nuclear and those localised on the mitochondrial DNA (mtDNA). There was no difference in the expression levels of genes involved in glucose metabolism and glycolysis with hexokinases, including *Hk3*, which initiates glucose metabolism; *Pfkb*, which aids in glycolysis regulation; and *Pklr,* which is involved in transphosphorylation to convert phosphoenolpyruvate into pyruvate and ATP at any time point. Taken together, these findings indicate that while there are differences in mitochondria-related genes, the amount of pyruvate needed for mitochondrial respiration may not differ.

In the cerebrum of both juvenile and adult mdx mice, there was a significant decrease in mtDNA gene transcripts compared with those in the cerebrum of age-matched controls and a significant increase in the mRNA levels of nuclear-encoded ETC subunits in 10d (*Ndufb8, Sdhb, ATP5a1*) and 10w (*Ndufb8*) mdx mice (Fig. 6a). Analysis of the mtDNA content revealed that while it increased with age in both genotypes, there was no difference in the mitochondrial to nuclear DNA ratio between the control and dystrophic cerebra (Fig. 6b). This increase in nuclear-encoded electron transport chain (ETC) subunit transcript levels in the cerebra of mdx mice was consistent with the increased protein levels of representative ETC proteins (Fig. 6c).

Additionally, GSMM revealed alterations in the reactions involved in mitochondrial respiration in 10d and 10w cerebra (Fig. 4, above). Specifically, the metabolic model indicated that the respiratory complex III coenzyme Q (cytochrome c—oxidoreductase; CYOR_u10mi), respiratory complex IV Cytochrome c oxidase subunit III (CYOOm3i) and ferrocytochrome-c: oxygen oxidoreductase reactions were upregulated at both ages. The upregulation of complex 4 was also observed in the 10w cerebra, as measured by western blot (Fig. 6c). Given the mitochondrial alterations in the GSM model, we next evaluated the expression levels of ETC genes and potential functional differences in the oxygen consumption rate (OCR) resulting from these changes in dystrophic cerebra.

The OCR values were measured using an Agilent Seahorse Analyser (XFe96) for isolated mitochondria after the administration of the mitochondrial uncoupler FCCP in three experimental variants: complex I (in the presence of complex I substrates but in the absence of succinate), complex II (in the presence of succinate and rotenone—a complex I inhibitor) and complexes I and II (in the presence of succinate and no rotenone).

The maximal mitochondrial respiration measured in the presence of the complex I and complex II substrates did not differ between the control and dystrophic cerebra. The interaction between age and genotype in the two-way ANOVA test was negative for all experimental variables. The maximal OCR values for complex I (Fig. 6d) showed an upwards but not statistically significant trend in mdx (10d animals: 21.84 ± 7.35 pmol O_2_/min/µg for C57BL/10 and 29.69 ± 22.10 pmol O_2_/min/µg for mdx; 10w animals: 33.45 ± 9.33 pmol O_2_/min/µg for C57BL/10 vs 45.42 ± 8.87 pmol O_2_/min/µg for mdx). However, an effect of age on the maximal OCR was observed for complex II (p value = 0.0254). The maximal OCRs in control animals were 22.15 ± 11.31 pmol O_2_/min/µg for 10d and 35.93 ± 6.17 pmol O_2_/min/µg for 10w C57BL/10, while they were 19.36 ± 11.85 pmol O_2_/min/µg for 10d mdx and 29.87 ± 9.67 pmol O_2_/min/µg for 10w mdx (Fig. 6e).

### Alterations of histone deacetylases (HDACs) in dystrophic cerebra

Given the upregulation of HDACs in dystrophic muscle and their therapeutic potential (Mercuri et al. 2024), we analysed these transcripts in the brains of mdx mice. HDAC5 and HDAC11 were consistently upregulated in the cerebra of 10d and 10w mdx mice. Additionally, in the 10d cerebra, HDAC6 and SIRT3 were overexpressed, while HDAC2, HDAC9, and SIRT1 were downregulated. No changes were observed in the dystrophic cerebella.

### The effects of full-length dystrophin loss in the cerebella are partially age dependent

**Fig. 7:**
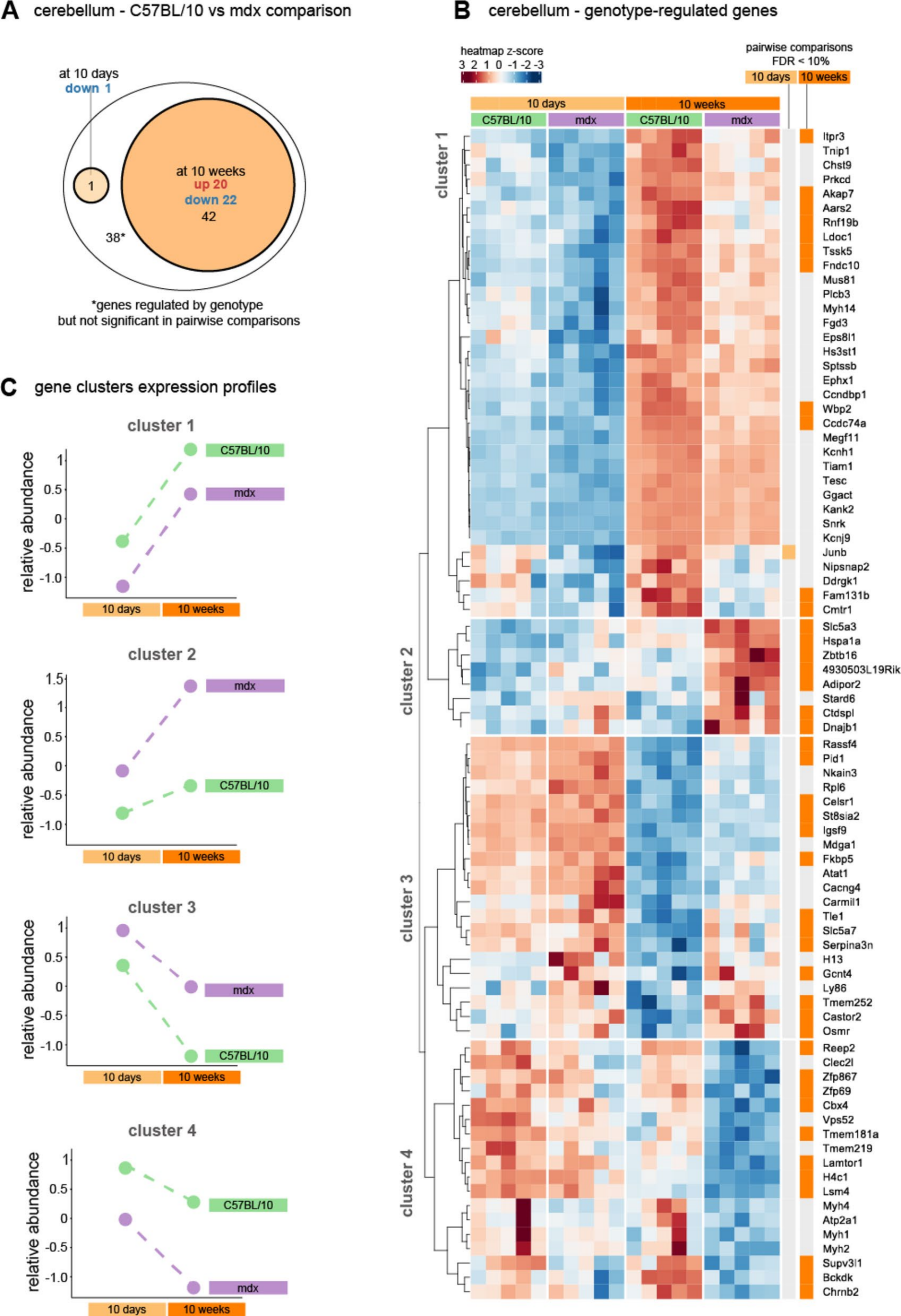
Differential gene expression in 10w and 10d mdx mice and C57Bl/10 cerebella. **A** Venn diagram showing the number of genes differentially expressed (FDR 10%) between mdx and C57Bl/10 mice. The largest ellipse contains all of the genes regulated by genotype (both ages considered together). The smaller circles show genes that were also significant in pairwise comparisons between mdx and C57Bl/10 mice at each age. **B** Heatmap of genes regulated in the cerebella at an FDR < 10% according to the genotype factor that did not show a significant gene‒age interaction (see Fig. 3). The intensity of the *coloured rectangles* represents transcript abundance levels. The presented level is proportional to the row z score values (between *darkest blue*: −3 and *darkest red*: 3), as displayed on the bar above the heatmap image. Hierarchical clustering was performed using correlation as a distance measure. The *light* and *dark orange bars* on the right of the heatmap highlight whether the gene is differentially expressed (FDR < 10%) in 10d and 10w mdx cerebella compared to age-matched controls. The full results are available in Supplementary Table 2. **C** Profiles of differences for the four identified clusters of altered genes; mean transcript abundance levels are presented for each group of genes

In the cerebella, 81 genes were differentially expressed between mdx and C57BL/10 mice at an FDR < 10%, but no significant interaction was detected between age and genotype (Fig. 7a; the full results are available in Supplementary Table 2). According to the pairwise comparisons, the majority of the regulated genes were differentially expressed between the 10w animals, and only one gene (*Junb*) was significantly differentially expressed at an FDR < 10% when the 10d animals were compared. Here, similar to those in the cerebra, four patterns of gene regulation according to age and genotype were identified (Fig. 7b, c). Clusters 1 and 2 included genes upregulated with age, and clusters 3 and 4 included genes downregulated with age. Genes from clusters 2 and 3 had higher expression in mdx animals, particularly in the 10w group. Genes in clusters 1 and 4 had lower abundances in mdx mice.

**Fig. 8:**
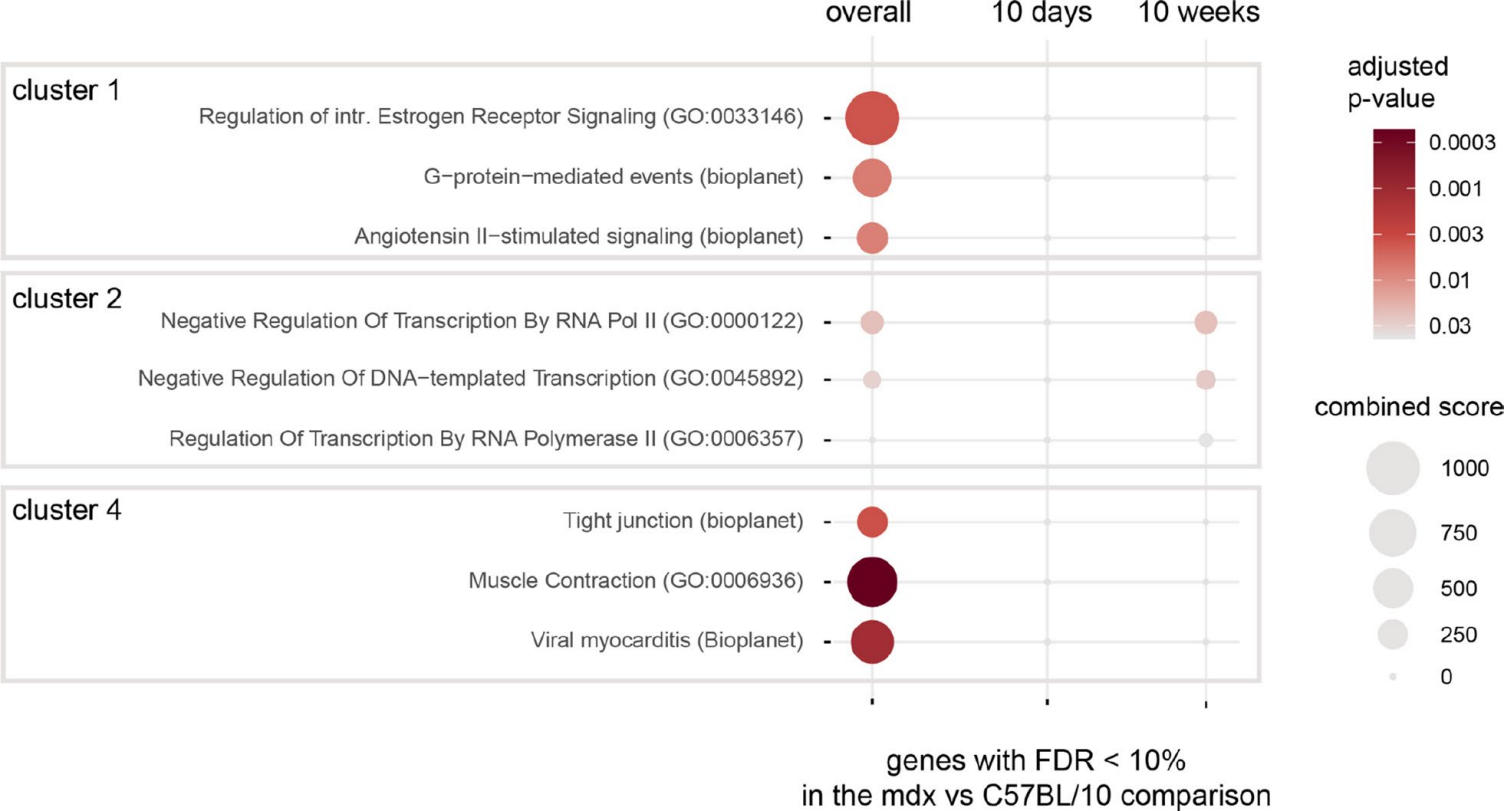
Biological processes and molecular pathways associated with genes differentially expressed in the cerebella of mdx mice. For each of the clusters, the top three enriched terms with at least two genes (ranked by adjusted *p* value) from the Bioplanet and GO Biological Process databases were selected. The full enrichment results are available in Supplementary Tables 5 and 6. The size of each circle reflects Enrichr’s combined score (calculated from the *p* value and odds ratio). The colour reflects the *p* value (the darker the colour is, the lower the *p* value). The first column shows the results from the full gene list from each cluster, while the second and third columns show the results for genes additionally filtered for significance in pairwise comparisons at each age. No enrichment was detected for cluster 3

Enrichment analysis revealed enriched GO and Bioplanet terms with at least 2 genes in clusters 1, 2 and 4 (Fig. 8). For each cluster, pathway enrichment was analysed for (1) overall genotype effect, (2) genes affected by genotype in 10d animals (only one gene—no enrichment was detected), (3) genes affected by genotype in 10w animals. Full gene lists from clusters 1 and 4 (decreased abundance in mdx mice) were enriched for terms associated with G protein-mediated signalling, including the angiotensin II and oestrogen receptor pathways and muscle contraction. This result was not significant if only genes with FDR < 10% in the pairwise comparison between 10w animals were used, signifying that this enrichment is evident only if all genotype-regulated genes are included. On the other hand, in cluster 2, the enrichment of genes involved in transcriptional regulation was also significant when only 10w animals were compared.

**Fig. 9:**
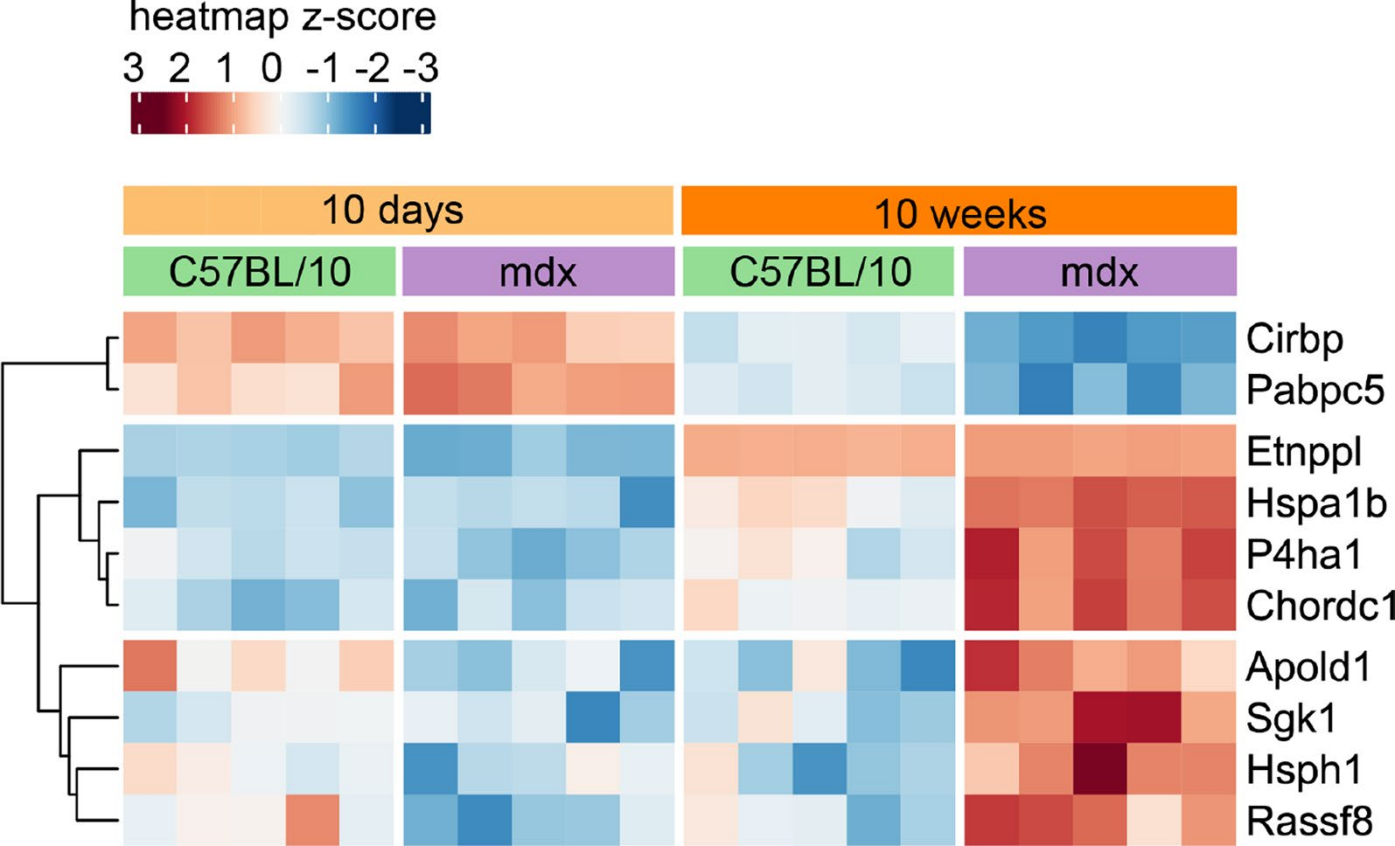
Age-dependent effects of full-length dystrophin loss in the cerebella. Heatmap of genes regulated in the cerebella according to age‒genotype interactions (FDR < 10%). The intensity of the *coloured rectangles* represents transcript abundance levels. The presented level is proportional to the row z score values (between *darkest blue*: −3 and *darkest red*: 3), as displayed on the bar above the heatmap image. Hierarchical clustering was performed using correlation as a distance measure to arrange the heatmap rows

Finally, in the cerebellum, we discovered ten genes whose expression was significantly differentially regulated by genotype depending on age (Fig. 9, age x genotype interaction FDR < 10%). The abundance of two of these genes, *Cirbp* and *Pabpc5*, markedly decreased in the 10w mdx group. *Cirbp* is a cold-inducible mRNA binding protein that acts as a transcriptional activator. *Pabpc5* plays a role in the regulation of mRNA metabolism in the cytoplasm. Eight genes were highly upregulated in 10w mdx animals, and this list included two members of the heat shock protein 70 family of proteins, the *Hsph1* and *Hspa1b* genes, as well as *Sgk1*, a robust regulator of gene expression involved in stress response regulation.

### Loco- and age-specific transcriptomic alterations in the dystrophic brain

RNA-seq studies of cerebral and cerebellar transcriptome changes revealed that at the chosen significance threshold, there were remarkably different levels of transcriptomic alterations between these two brain regions. The observed difference may be caused by several contributing factors. The first possible explanation is that the effects of mdx mutations in the cerebra are indeed global, while cerebellar effects are subtler. Second, the difference in developmental trajectories between the two brain regions may have contributed to the observed results. It is important to note that, unlike in the cerebrum, extensive cerebellar development in humans occurs postnatally, continuing into the early teens, and in 10d mice, the cerebellum is not fully mature (White and Sillitoe 2013). Hence, the molecular changes resulting from the loss of full-length dystrophin may signify brain region-specific deficiencies compounded by maturation-dependent alterations in functionality. This explanation is supported by the fact that for each tissue, regardless of the genotype, more than 10 000 transcripts were differentially expressed between 10d and 10w animals (FDR < 10%, see full statistical results in Supplementary Tables 1 and 2). Lastly there is a possibility that cerebellar transcripts show greater variability; therefore, the analysis of the cerebellum is underpowered compared to that of the cerebrum. To evaluate this factor, we analysed the common biological coefficient of variation (BCV), which is a parameter that estimates how the true abundance of genes varies between replicate samples under the negative binomial gene expression model assumed in the analyses here. The BCV represents the variation that would remain between biological replicates if the sequencing depth could be increased indefinitely (https://www.ncbi.nlm.nih.gov/pmc/articles/PMC2796818/). Indeed, the overall BCV was approximately 60% greater in the cerebella (Table 2), which was driven mainly by the cerebella of younger animals of both genotypes. This observation is consistent with the immaturity of 10d cerebella.

**Table 2:** Biological coefficient of variation estimates for the analysed transcriptomes of BCVs were computed using the R edgeR library (https://www.ncbi.nlm.nih.gov/pmc/articles/PMC2796818/)

Tissue	C57BL/10 10 days	C57BL/10 10 weeks	mdx 10 days	mdx 10 weeks	Overall
Cerebrum	0.098	0.108	0.104	0.149	0.116
Cerebellum	0.181	0.108	0.283	0.14	0.187

We also observed that the changes in gene expression with age had opposite trajectories in the cerebra and cerebella. In the dystrophic cerebra, more DEGs were identified at 10 days than at 10 weeks, which was the opposite for the cerebella. Additionally, no genes showed a significant age‒genotype interaction in the cerebra, while in the relatively small set of DEGs in the cerebella, there were 10 genes for which this interaction was significant (FDR < 10%). This shows that in the cerebella, the effects of the mdx mutation are more age-dependent than they are in the cerebra.

**Fig. 10:**
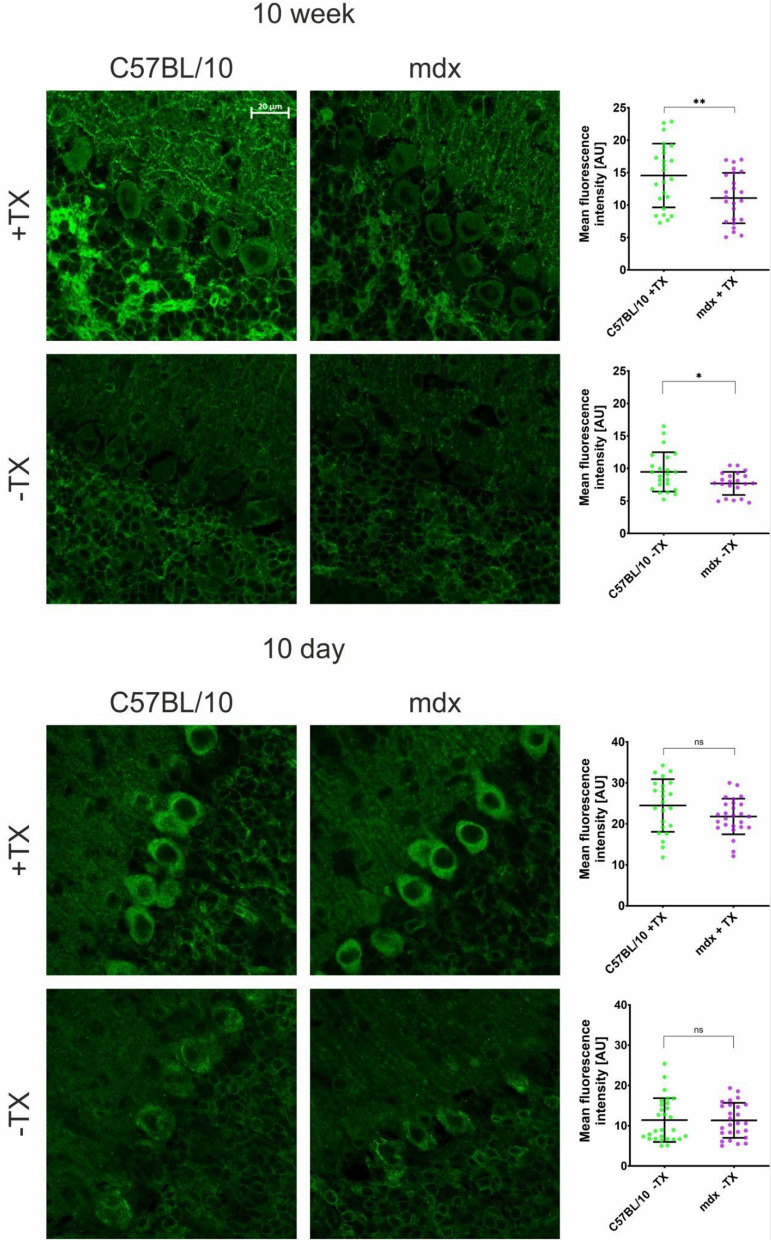
The immunoreactivity of GABAAR α1 in 10w and 10d cerebella. Representative immunofluorescence images of samples with (+TX) and without (−TX) Triton X-100 permeabilization are shown alongside enumerations of the corresponding fluorescence intensity within the Purkinje neuron layer. The quantification of the immunofluorescence intensity is shown as the mean and standard deviation (SD) of 23–29 individual images showing 112–154 Purkinje neurons. *p < 0.05 unpaired Student’s *t* test, *ns* no significant difference. *Scale bar* = 20 µm

To further investigate whether dystrophic abnormalities may occur late in the cerebellar maturation process, we analysed the synaptic clustering of GABAA receptors, whose alteration in the cerebella of mdx mice is a well-established dystrophic abnormality (Knuesel et al. 1999; Zarrouki et al. 2022). Both the total GABAAR α1 level and plasma membrane-associated GABAAR α1 localization were affected by *Dp427p* loss in the cerebella of 10w mdx mice (Fig. 10). In contrast, in 10d mice, no difference in GABAAR levels or distribution was found in dystrophic cerebella, which confirmed the transcriptomic finding of the paucity of early developmental abnormalities in the absence of full-length dystrophin.

### Dystrophic abnormalities in the cerebellum

**Fig. 11:**
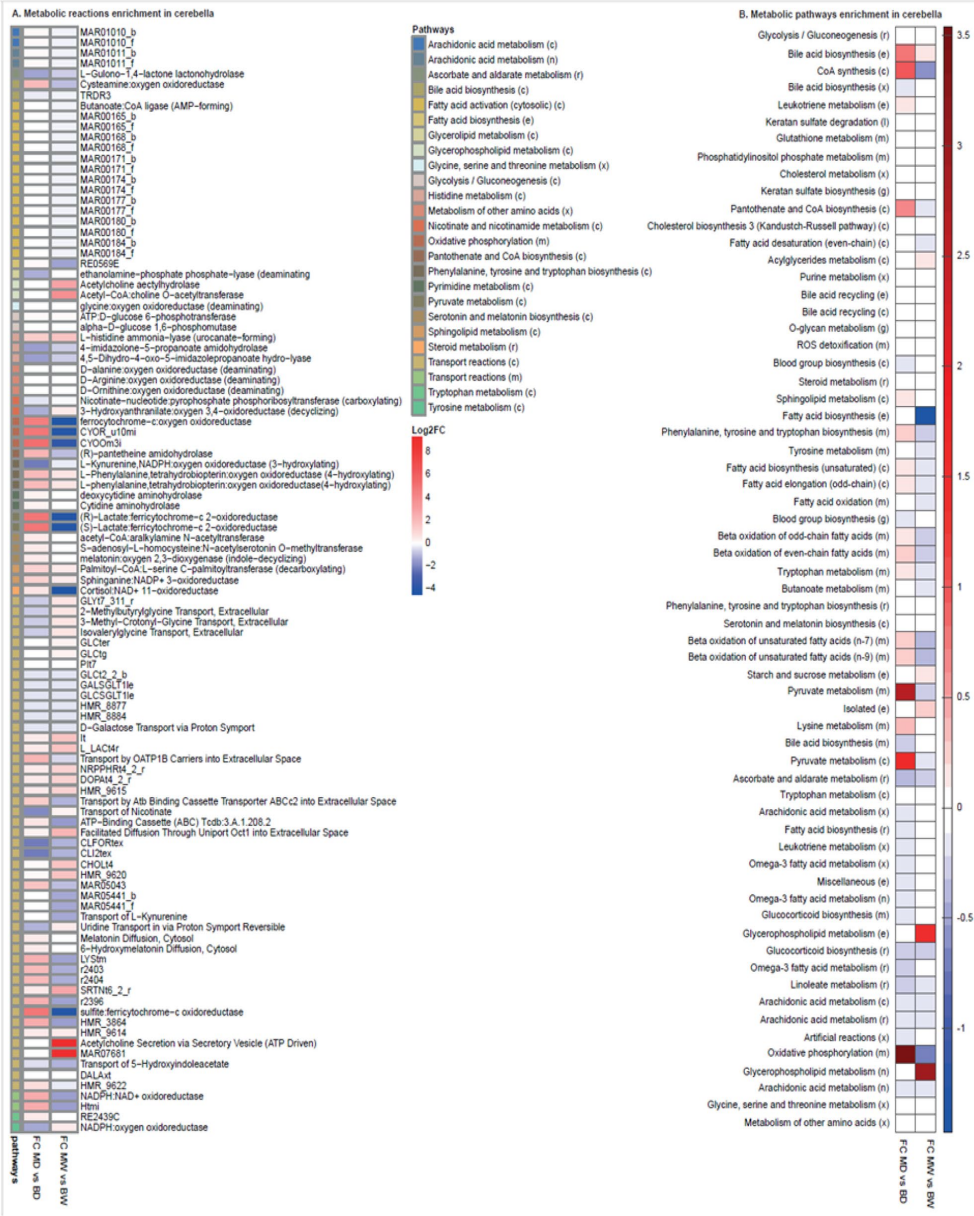
Reaction enrichment analysis in cerebella. **A** Reaction flux fold changes between mdx and C57Bl/10 for 10d and 10w were estimated. The reactions are sorted by pathways, the pathways associated with each reaction indicated; **B** Pathways flux fold change between mdx and C57Bl/10 cerebella for 10d and 10w. The reactions are grouped by pathways, indicating the pathways associated with each reaction. For **A** and **B**, the upregulated and downregulated reactions are shown in *red* and *blue*, respectively; “_b” and “_f” represent backwards and forward reactions, respectively. Compartments: (e): Extracellular, (x): Peroxisome, (m): Mitochondria, (c): Cytosol, (l): Lysosome, (r): Endoplasmic reticulum, (g): Golgi apparatus, (n): Nucleus, (i): Inner mitochondria

The mitochondrial respiratory-associated reactions in the cerebella were analysed in an analogous manner to those in the cerebra (Fig. 11), and this modelling indicated that the respiratory complex III coenzyme Q (cytochrome c—oxidoreductase CYOR_u10mi), complex IV Cytochrome c oxidase subunit III (CYOOm3i) and ferrocytochrome-c: oxygen oxidoreductase reactions might be downregulated in the 10w cerebella of mdx mice. The decreased activity of complex III and IV respiratory reactions agrees with previous findings (Tuon et al. 2010). Transport reactions (GLCt2_2, GLCSGLT1le, and GLCt4_2) involved in glucose metabolism were downregulated in the cerebella of mdx mice at both ages.

**Fig. 12:**
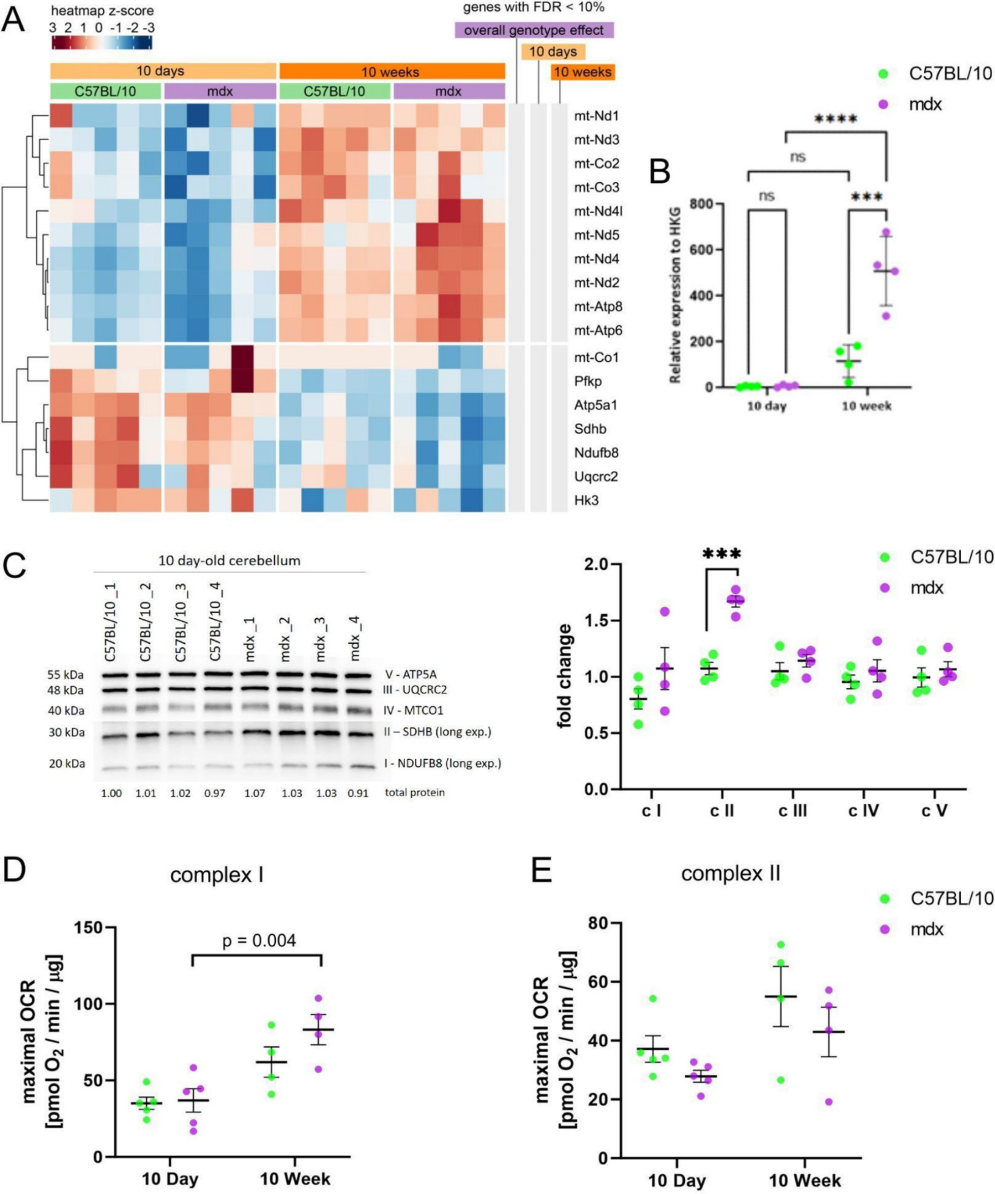
Mitochondrial alterations in 10d and 10w mdx cerebella due to full-length dystrophin loss. **A** Heatmap of the abundance of selected ETC genes in the cerebella. The intensity of the *coloured rectangles* represents transcript abundance levels. The presented level is proportional to the row z score values (between darkest blue: −3 and darkest red: 3), as displayed on the bar above the heatmap image. To order rows (genes), hierarchical clustering was performed using correlation as a distance measure. The annotations in columns on the right of the heatmap highlight whether the gene is differentially expressed (FDR < 10%) according to the global genotype effect and then in 10d and 10w mdx cerebella compared to age-matched controls. All of the genes apart from Uqcrc2 were also differentially expressed between ages (FDR 10%). **B** The ratio of mitochondrial to nuclear DNA (mtDNA/nDNA) in the cerebella of 10d and 10w C57Bl/10 and mdx mice. Two-way ANOVA with Tukey’s post hoc test was performed. *NS* not significant, ***p value < 0.001, ****p value < 0.0001. **C** Protein content of ETC subunits in 10d cerebella from C57Bl/10 and mdx mice. The ETC subunits NDUFB8 (complex I; c I), SDHB (complex II; c II), UQCRC2 (complex III; c III), MTCO1 (complex IV; c IV) and ATP5A (complex V; c V) were detected by western blot analysis and normalized to total protein in lines visualized with Ponceau S. Normalization factors are shown under the representative western blot images. The charts present the mean values with standard deviations. Statistical significance was determined with Student’s t test; n = 4; ***p < 0.001. The maximal oxygen consumption rate (OCR) of mitochondria isolated from C57Bl/10 and mdx cerebella at 10d and 10w after the administration of FCCP for **D** complex I (in the absence of the complex II substrate succinate) and **E** the complex I inhibitor rotenone and complex II (in the presence of succinate and rotenone). Two-way ANOVA was performed together with multiple comparisons *t*-tests with Bonferroni correction. The charts present the mean and SD values. The number of animals included in the analysis was 5 for 10d and n = 4 for 10w animals

In contrast to those in the cerebrum, no differences in mtDNA gene transcript or nuclear-encoded mitochondrial transcript expression were found in the cerebellum (Fig. 12a). Although the ratio of mitochondrial DNA to nuclear DNA in the cerebella of 10w mdx mice was greater than that in the cerebella of 10w C57Bl/10 mice (*p* = 0.0001) (Fig. 12b), Western blot analysis revealed no difference in the ETC subunit levels between 10w mdx and C57Bl/10 mice (Supplementary Fig. 5). Interestingly, the ETC complex II protein was significantly upregulated in 10d mdx cerebella (*p* = 0.0002) (Fig. 12c).

Potential differences in the oxygen consumption rate were also investigated functionally in mitochondria isolated from control and dystrophic cerebella (Fig. 12d, e). Two-way ANOVA revealed an age-related effect on the maximal OCR for all experimental variants (*p* value = 0.0004 for complex I, *p* value = 0.023 for complex II and *p* value = 0.044 when substrates for both complexes were present together), while in the cerebra, the age effect was observed only for complex II.

The OCR for complex I increased with age in both groups (OCRs: 35.07 ± 9.07 and 61.93 ± 19.72 pmol O_2_/min/µg for 10d and 10w C57Bl/10, respectively and 36.93 ± 17.11 and 83.22 ± 19.75 pmol O_2_/min/µg for 10d and 10w mdx, respectively; Fig. 12d). The maximal OCRs for complex II in control animals were 37.16 ± 10.06 and 54.99 ± 20.42 pmol O_2_/min/µg for 10d and 10w C57Bl/10 mice and 27.86 ± 4.52 and 42.94 ± 16.82 pmol O_2_/min/µg for 10d and 10w mdx mice, respectively (Fig. 12e). However, multiple comparisons tests revealed no significant differences (*p* value > 0.05).

Unlike in the cerebra, no change was observed in the GSMM-predicted glycolysis/gluconeogenesis pathway in the mdx cerebella. Additionally, the loss of full-length dystrophin did not affect the expression levels of the enzymes involved in glucose metabolism (*Pfkp*, *Hk3*, *Pkir*; Fig. 12a), nor did it impact the protein levels of the glucose transporter Glut1, as assessed by Western blotting (Supplementary Fig. 6).

### mRNA splicing alterations in dystrophic cerebra and cerebella

**Fig. 13:**
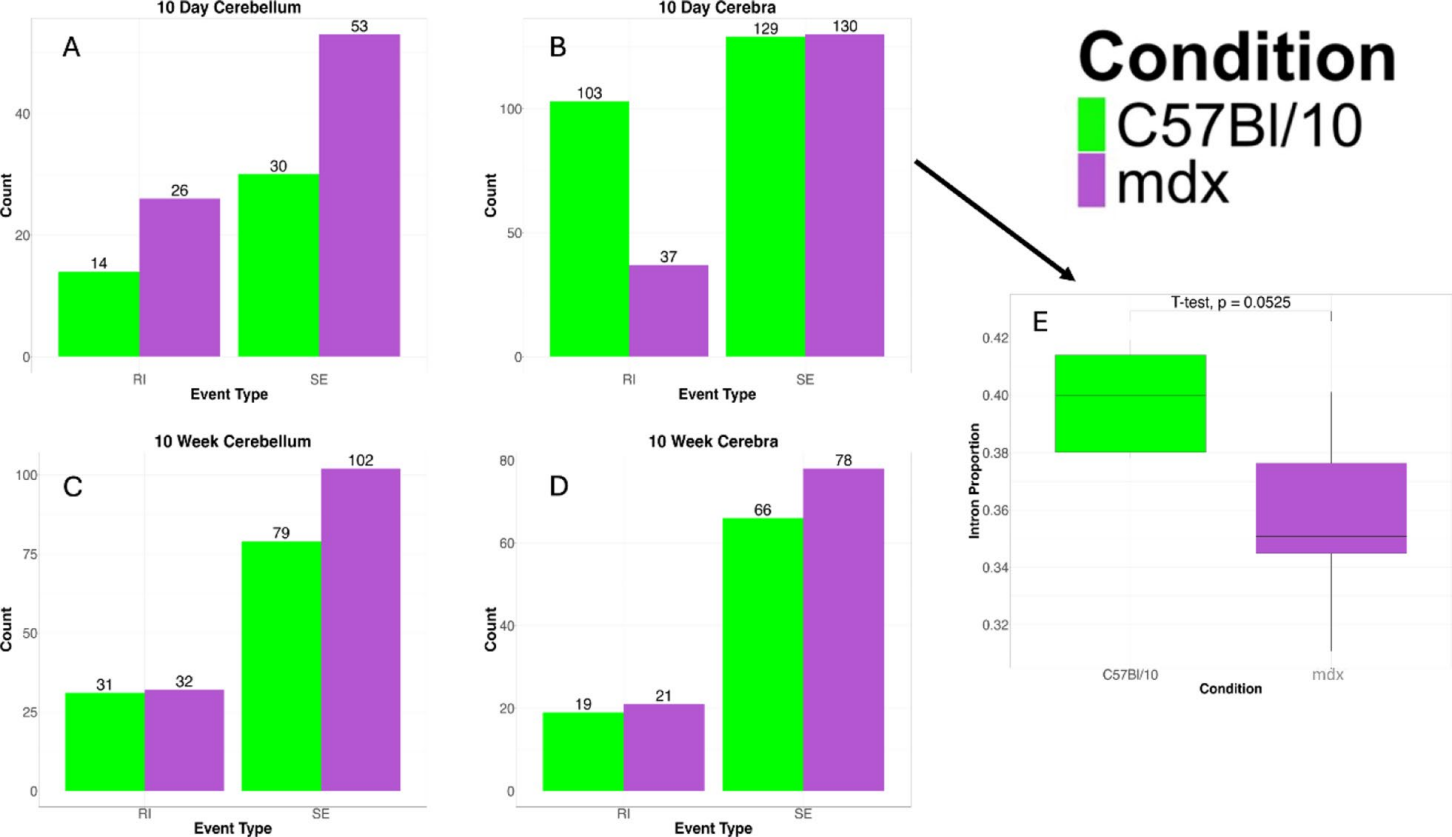
Differential alternative splicing events in the cerebella and cerebra of mdx mice. **A**10 day cerebellum, **B** 10 day cerebrum, **C** 10 week cerebellum, **D** 10 week cerebrum. The numbers refer to events that are more pronounced in a given brain region and timepoint. Overall, there was a tendency toward more skipped exons and retained introns in mdx mice. **E** There was a significant reduction in the number of intron retention events (*p* = 2*10^−8^, sign test) in the cerebra of 10d mdx mice, which was accompanied by an overall genome-wide reduction in the proportion of intronic reads. *RI* retained introns, *SE* skipped exons

RNA splicing was an enriched pathway identified in dystrophic cerebra at 10 days and 10 weeks (Fig. 3, cluster 2), and the genes encoding heterogeneous nuclear ribonucleoproteins (HNRNPs) were particularly downregulated (*Hnrnpa2b1, Hnrnpa3, Hnrnpab, Hnrnpdl, Hnrnph3, Hnrnpll, Hnrnpr, Hnrnpu, Hnrnpul2*), in addition to *Srsf4, U2af2, Prpf3, Prpf39* and *Fus*. Therefore, we analysed potential splicing abnormalities in dystrophic brains. Overall, in the mdx mouse brain, we observed a propensity toward increased exon skipping (SE) and intron retention (RI) in nearly all conditions (Fig. 13). This finding is consistent with generalized splicing defects in mdx mice. The 10d cerebrum was a striking outlier of this trend: mdx mice exhibited a significantly reduced number of retained introns (Fig. 13b), which was also reflected in a reduced genome-wide proportion of intronic reads in the RNA-seq data (Fig. 13e), suggesting a general trend toward more efficient intron splicing. The high number of differential splicing events in the 10d cerebral region coupled with the high number of DEGs detected further suggested that the 10d cerebral tissue is particularly affected by the loss of dystrophin. The overall decrease in the number of detected alternative splicing events between 10d and 10w in the cerebra aligns with the nonprogressive nature of neuropsychiatric abnormalities in DMD.

In 10d cerebra, more RI events were detected in the control than in the mdx group (103 vs. 37). More than 30% of the total differential alternative splicing events in the wild-type were RI events, compared to approximately 17% in the mdx. No other events were significantly different between the control and mdx groups in the 10d cerebra. At 10 weeks in the cerebra, there was a slight difference in the SE, with 66 detected in C57Bl/10 and 78 detected in mdx mice.

At 10 days in the cerebella, more RI events were detected in the mdx mice than in the control mice (14 vs. 26). More SE events were also detected in mdx mice (30 vs. 53). Notably, while the cerebra had far fewer events at 10w than at 10d in both wildtype and mdx mice, more events were detected at 10w than 10d cerebella. More differential SE events (79 vs. 102) were also detected in the cerebella of the mdx mice at 10w than in those of the control mice. Overall, the differential alternative splicing in the cerebella was less dramatic than that in the cerebra. However, the contrast between the trajectories of differential alternative splicing trajectories for these two tissues is notable.

### Neuroinflammation in dystrophic cerebra and cerebella

Next, we evaluated the potential impact of inflammatory mediators on dystrophic dysfunction by comparing 10-day-old and 10-week-old dystrophic brain regions. Analysis of transcripts typically associated with neuroinflammation, astrogliosis, microgliosis, activated microglia and brain-infiltrating macrophages (DePaula-Silva et al. 2019) revealed no consistent upregulation in 10w dystrophic cerebra and cerebella (Supplementary Figs. 7, 8, Supplementary Tables 7,8). Interestingly, several of these transcripts were differentially expressed already in 10d cerebra, namely *Trem2, Cx3cl1* and *Ngf* (upregulated), and *Bdnf* and *Il18* downregulated in mdx.

In the pathway analysis, we found the Response to Reactive Oxygen Species pathway to be enriched in 10d and 10w mdx cerebra and present in clusters 1, 3, and 4 in the dystrophic cerebra (Supplementary Table 3). Clusters 1 and 4 contain genes that are upregulated in mdx cerebra and cluster 3 contains genes that are downregulated in mdx cerebra. No classical inflammatory pathways were enriched in DEGs in the cerebra of 10w mdx mice. However, we detected a decrease in the expression of kynurenine aminotransferase 1 (*Kyat1)* in the cerebra of 10-day-old mdx. There was a decrease in the production of L-kynurenine, which was an enriched pathway indicated by GSMM in 10-day-old mdx cerebra and cerebella. This indicates that young mdx mice have lower levels of circulating kynurenic acid, a metabolite of L-kynurenine with a neuroimmunoregulatory role (Mithaiwala et al. 2021). This agrees with a previous study (Copeland et al. 2022).

Given the significant impact of P2X7 purinoceptor ablation on cognitive and behavioural functions in mdx mice (Sinadinos et al. 2015), we studied the expression of this key inflammatory trigger. There was no significant difference in the expression of any of the two mouse transcript variants (P2X7a and P2X7k) assessed by qPCR (Supplementary Fig. 9) or in the P2X7 protein levels in 10d or 10w cerebra compared to age-matched controls (Supplementary Fig. 10). There was also no difference in the expression of P2X7a, b, or c 3′-nd splice variants, as assessed by PCR, in brain regions at any time point (Supplementary Fig. 11).

**Fig. 14:**
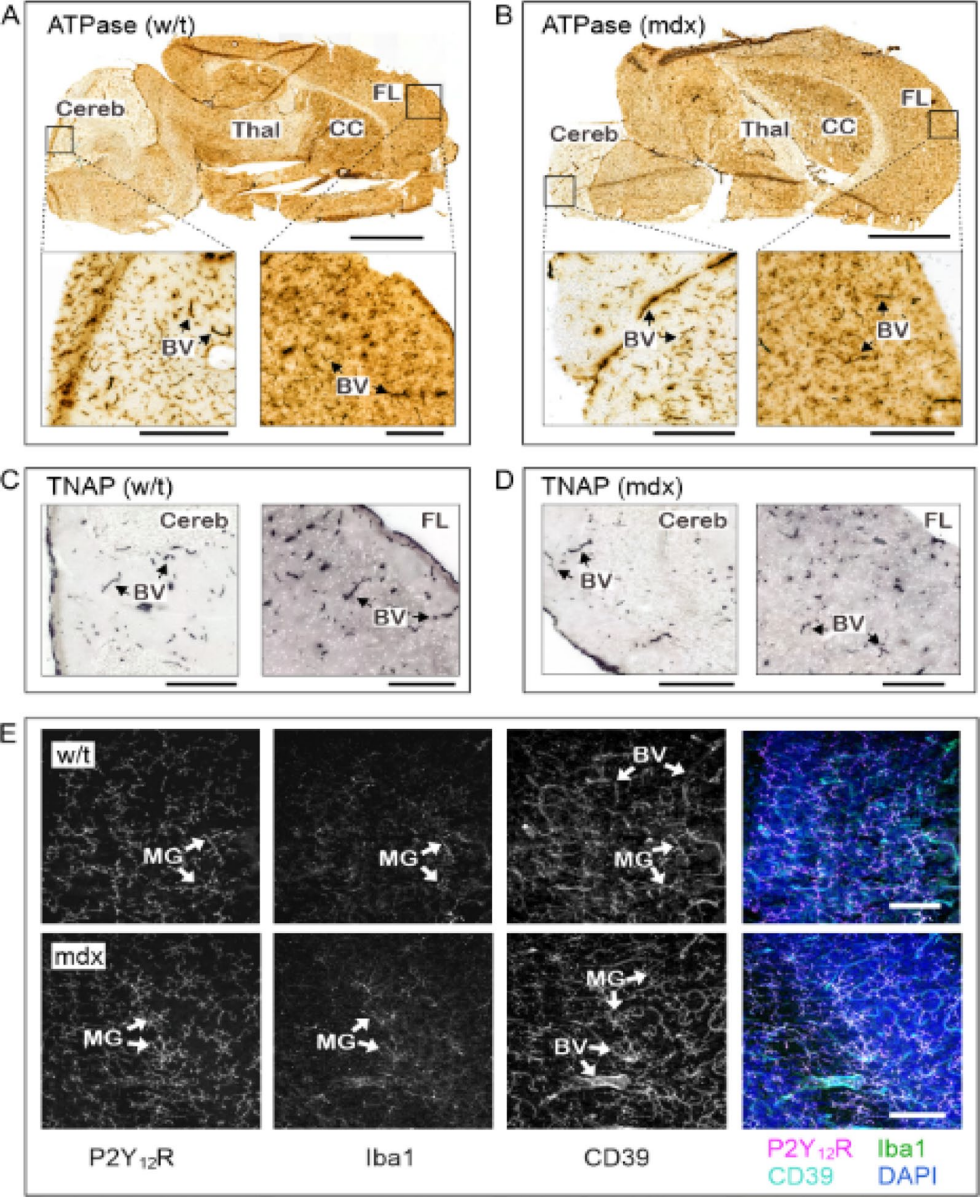
Histochemical analysis of the distribution of blood vessels and microglia in C57Bl/10 (w/t) and mdx brain ATPase/CD39 activity was performed in situ by incubating cryosections of **A** w/t and **B** mdx mouse brains with 300 μM ATP in the presence of Pb(NO_3_)_2_, followed by microscopic detection of ATP-derived inorganic phosphate as a brown precipitate. TNAP activity was measured in **C** w/t and **D** mdx mouse brains using the chromogenic substrates BCIP and NBT and subsequent monitoring of the development of blue colour reactions. **E** LMA-embedded brain sections from w/t (*upper panels*) and mdx (*lower panels*) mice were stained for the molecular microglia markers Iba1, P2Y12R and CD39 (with the latter enzyme also co-expressed on vascular endothelial cells), as indicated. 3D images of 100 µM tissue were captured using a spinning disk confocal microscope. Maximum intensity projections for each channel are shown in grayscale, with the right panels displaying merged images with nuclei counterstained with DAPI. *BV* blood vessel, *CC* corpus callosum, *Cereb* cerebellum, *FL* frontal lobe, *MG* microglia, *Thal* thalamus. *Scale bars*: 2 mm (**A**), 300 μm (*insets* in **A**, **B**), and 80 μm (**E**)

Next, we evaluated the expression level and enzymatic activity of ectonucleoside triphosphate diphosphohydrolase-1 (NTPDase1, CD39) in the mdx mouse brain. This enzyme governs the duration and magnitude of purinergic responses via rapid inactivation of extracellular ATP and ADP (Yegutkin 2008). The tissue-specific distribution of CD39 activity was determined in situ by using a lead nitrate-based enzyme histochemistry assay (Langer et al. 2008; Losenkova et al. 2020). Incubation of brain cryosections from wild-type (Fig. 14a) and mdx (Fig. 14b) mice with ATP as a preferred substrate for CD39 revealed substantial ATPase activity in the frontal lobe, cerebellum and other brain areas, with the most intense staining detected in the brain vessels and capillaries. We also measured the catalytic activity of another ectoenzyme, tissue-nonspecific alkaline phosphatase (TNAP), by using a mixture of the artificial chromogenic substrates BCIP and NBT. The highest TNAP activity was detected in the blood vessels of various calibres, with no differences detected between the wild-type (Fig. 14c) and mdx (Fig. 14d) brains. Taken together, a similar pattern of blood vessel-specific localization of CD39 and TNAP in wild-type and mutant brains did not confirm gross vascular abnormalities in blood‒brain barrier integrity in mdx mice, which disagrees with previous studies (Nico et al. 2003, 2004).

The inflammatory state of the mdx mouse brain was also assessed by measuring the distribution and stereoscopic morphology of microglia by staining for the following molecular markers: P2Y_12_ receptor (P2Y12R), ionized calcium binding adaptor molecule-1 (Iba1), and CD39 (with the latter ectoenzyme also being co-expressed on the vascular endothelium). No significant differences in microglial distribution, morphology or activation state (CD39^+^/P2Y_12_R^+^/Iba1^+^) were found between 10w cerebra from mdx and matched control mice (Fig. 14e). Similarly, there was no difference in the expression levels of the genes (Aif1 and P2y12r) encoding these microglial markers.

## Discussion

An impact on cognitive function is observed in all DMD patients, although the severity of this effect varies greatly (Banihani et al. 2015; Cotton et al. 2001). When severe, it can significantly worsen the clinical features of this debilitating disease. However, our understanding of the underlying molecular alterations remains largely incomplete. The genotype‒phenotype correlation partially explains the differences between patients with proximal versus distal mutations affecting full length or all dystrophins, respectively. However, the majority of DMD patients have mutations affecting full-length isoforms only, yet these patients still exhibit cognitive and behavioural impairments of varying severity. Most of these isoforms are expressed in specific brain regions, and some of their roles have been identified (Abdulrazzak et al. 2001; Górecki et al. 1992; Lidov 1996). However, their functional relationships with cognition and behaviour remain unclear. Furthermore, cognitive impairment is generally considered to start in development, with full-length dystrophin having the highest expression in humans at 2 years (Doorenweerd et al. 2017) and being nonprogressive; however, its evolution from the perinatal stage through adolescence to adulthood has not been studied in detail. Finally, the potential impact on cognitive functions of the dystrophic BBB (Frigeri et al. 2001; Nico et al. 2003) and of inflammatory mediators assaulting the brain through it (Greene et al. 2024) have not been investigated.

We attempted to identify the key factors underlying these neuropsychiatric abnormalities using a combination of transcriptomic and functional analyses in the mdx mouse model of DMD. Our data revealed that the absence of full-length dystrophin transcripts leads to a wide range of abnormalities, yet no common defects were observed between brain regions at specific time points. Transcriptomic brain alterations resulting from the loss of full-length dystrophin expression are both region-specific and age-dependent and are compounded by maturation/adaptation-dependent alterations.

The effects of the mdx mutation on the cerebella appear particularly age dependent, likely reflecting the immaturity of 10d cerebella. It is important to note that the cerebellum is not fully developed in 10-day-old mice. Thus, the molecular alterations resulting from the loss of full-length dystrophin mostly occur after functional maturation, which occurs at approximately postnatal day 15 (White and Sillitoe 2013). This paucity of early cerebellar symptoms extends to well-known anomalies, such as GABAA receptor clustering (Fritschy et al. 2012; Krasowska et al. 2014; Zarrouki et al. 2022), which we found to be unaltered in 10d cerebella. Thus, the functional consequences of altered GABAergic synapses (Kreko-Pierce and Pugh 2022; Snow et al. 2014; Stay et al. 2019; Suzuki et al. 2017) are also likely to be late symptoms. As such, timely treatment could reverse or reduce this impact.

Although no compensatory overexpression of the short isoforms was detected, in line with previous studies in mouse and canine models (Crawford et al. 2023; Stay et al. 2019), there was significant cerebellar expression of Dp427c in addition to Dp427p expressed in Purkinje cells only (Górecki et al. 1992), which is also in agreement with previous findings (García-Cruz et al. 2023). Therefore, cerebellar alterations cannot be solely attributed to occurring in this one cell type. The simultaneous expression of Dp427c and Dp427p transcripts can be excluded not only on the basis of in situ hybridization data (Górecki et al. 1992) but also because the conflicting steric demands preclude such expression from a single *DMD* locus available for transcription within a cell (Hildyard and Piercy 2023). Although the cellular origin of Dp427c in the cerebellum is currently unknown, some can be considered. The choroid plexus was shown to express Dp427 in the developing canine cerebella (Hildyard et al. 2020), but given the anatomical localization in relation to the adult cerebellum, the choroid plexus is less likely to be the source. Garcia-Cruz et al. (2023) reported that Dp427c mRNA was found not only in neurons but also in astrocytes and the OPC.

While some DEGs in dystrophic cerebella (Tanc2 (upregulated), Flrt3, Reep2, Rnf19ab, and Rnps1 (downregulated)) are expressed in Purkinje cells, cell enrichment analysis performed as described in Kozareva et al. (2021) did not conclusively identify particular cell type involvement. Of note, The Rnps1 encoded protein is involved in mRNA surveillance (Mabin et al. 2018; Viegas et al. 2007), and its downregulation could potentially impact nonsense-mediated mRNA decay (Viegas et al. 2007) and splicing alterations described below.

In contrast to those in the cerebella, in 10d mdx cerebra over a quarter of all genes were differentially expressed (6314). These DEGs reflect combined alterations in the hippocampus, amygdala, cortical neurons and other cells, such as astrocytes, which are known to express full-length dystrophins (Patel et al. 2019). These DEGs likely reflect changes caused by the loss of dystrophin expression and those induced by various compensatory mechanisms activated in the young dystrophic brain. Interestingly, there was a fourfold reduction in the number of DEGs at 10 weeks, indicating that the majority of dystrophic abnormalities and compensatory changes in the cerebrum occur during the prenatal and early postnatal periods. However, Bagdatlioglu et al. (2020) reported a deterioration in short- and long-term memory in mdx mice aged from 4 to 12 months (Bagdatlioglu et al. 2020). Clearly, the progressive vs. nonprogressive nature of DMD brain abnormalities requires further study.

The mechanism leading from the loss of full-length dystrophin to a high percentage of DEGs is not clear. One explanation could be the regulatory role played by this protein in transcription. For example, in dystrophic myoblasts, the significantly downregulated expression of *Myod1* corresponded with the majority of downregulated genes being controlled by this transcription factor (Gosselin et al. 2022). However, here, TFBS analysis did not produce a clear result. However, this is rather unsurprising given the diversity of cells expressing dystrophin in these brain regions compared with the single myoblast expression profile.

Interestingly, in both 10d and 10w cerebella, we detected more differential alternative splicing events in the mdx mice. However, while the cerebra had far fewer events at 10w compared to 10d, in cerebella more events were detected at 10w than 10d. In the cerebella at both timepoints, as well as in the 10w cerebra, there was a general trend toward more splicing events in mdx mice than in the controls. The 10d cerebrum was, however, a striking outlier of this trend: while a similar number of SE events was detected between the two genotypes, the mdx exhibited a significantly reduced number of retained introns. This was also reflected in a reduced genome-wide proportion of intronic reads in the RNA-seq data, suggesting a general trend towards more efficient intron splicing. This observation seems counterintuitive in a disease model because intron retention has been linked to diseases (reviewed in Kumari et al. 2022). In fact, it was connected to a lack of dystrophin expression in rhabdomyosarcoma cells (Niba et al. 2017). However, during normal development, intron retention is an important regulatory mechanism (Jacob and Smith 2017). It may represent an element of the tissue-specific process of functional tuning of the transcriptome, where it restricts protein translation only to cells that require it while maintaining transcription from the same locus in other tissues. This finding is particularly interesting given that several unusual, developmentally regulated *Dmd* splice variants have been found in mouse and human brains. These mRNAs with inclusion of intronic sequences such as cryptic exons or pseudoexons are translated into proteins (García-Cruz et al. 2023). Thus, the reduction in intron retention may in fact represent a regulatory defect. Notably, if DMD causes random splicing aberrations that trigger nonsense-mediated RNA decay, this could explain the large number of DEGs and the high variability in alterations between brain regions and timepoints.

Dysregulated splicing in DMD brains is a completely new finding. This discovery is also particularly interesting given that abnormal splicing has emerged as an aetiology of major neurological diseases (Nikom and Zheng 2023) and can affect neurodevelopment (Li et al. 2024). Similarly, the large number of downregulated heterogeneous nuclear ribonucleoproteins (hnRNPs) involved in alternative splicing, transcriptional and translational regulation, stress granule formation, cell cycle regulation, and axonal transport might contribute to DMD-related neurological dysfunction, as has been shown for other neurological diseases (reviewed in Low et al. 2021).

Similarly, altered expression of HDACs, enzymes regulating the deacetylation of histone and non-histone proteins, affects a wide range of cellular processes. Interestingly, HDAC abnormalities have been found in DMD muscle (Sandonà et al. 2023), and here, HDAC5 and HDAC11 were also consistently upregulated in the dystrophic cerebra. Thus, the absence of dystrophin causing HDAC upregulation could explain the numerous alterations in the expression of a wide range of genes occurring across such different tissues. Interestingly, HDAC5 and HDAC11 have been linked to a range of functions, including cognition and neuroinflammation (Gräff et al. 2012; Kumar et al. 2022), which are notably altered in DMD. Given that HDAC inhibitors have improved muscle abnormalities in clinical trials, their potential to mitigate neuropsychiatric defects should be explored.

Carbohydrate catabolism was another pathway alteration in the cerebra. Brain energy demands are covered by the glucose supply from the blood, which is evoked by glucose transporters (GLUTs), which mediate glucose uptake across the BBB (Koepsell 2020). These transporters are critically involved in regulatory adaptations to varying energy demands in response to differing neuronal activities and glucose supplies. Mutations in *Slc2a1* cause GLUT1 deficiency syndrome and cognitive impairment (Giorgis et al. 2019). Interestingly, there was a reduction in GLUT1 levels in 10d dystrophic cerebra but not at 10 weeks. However, the trend showed that there might be greater expression in 10w mdx cerebra given the much bigger fold increase in mdx compared to C57Bl/10 between 10d and 10w (Fig. 5). This GLUT1 normalization is in line with previous data showing that GLUT1 levels in 6–7-month-old mdx and control mice were similar (Olichon-Berthe et al. 1993). The reduction in GLUT1 in 10-day-old dystrophic cerebra also aligns with the reductions in the activity of metabolic reactions, we identified. Given that GLUT1 is present in the BBB, alterations in the dystrophic BBB (Nico et al. 2004; Uchida et al. 2011) may involve a reduction in glucose transport.

Mitochondrial alterations arise early in the pathophysiology of DMD, with mitochondrial gene dysregulation at the somite (Mournetas et al. 2021), dysfunctional mitochondria present in DMD myofibres and myoblasts, impaired mitophagy, and altered energy metabolism found in these cells (Moore et al. 2020; Onopiuk et al. 2009; Rybalka et al. 2014; Scholte and Busch 1980; Sebori et al. 2018; Tracey et al. 1995). A previous study of cortical brain slices from old (>6 months) mdx and control mice revealed no differences in oxygen consumption in any metabolic setting (glucose, pyruvate or b-hydroxybutyrate/acetoacetate) or any differences in glucose uptake. However, increased substrate-dependent oxygen consumption rates at low oxygen partial pressures were observed, suggesting greater susceptibility of the mdx mouse brain to ischemia (Rae et al. 2002).

Alterations in mitochondrial metabolism are also among the earliest anomalies found in the mdx mouse brain here, with an increase in complex 2 protein expression in dystrophic cerebella at 10d (Fig. 12c). This occurs prior to functional cerebellar maturation (White and Sillitoe 2013). During development, mitochondrial functions increase, with more alterations observed in the dystrophic cortex at 10w than 10d. This finding is in line with oxygen consumption rate (OCR) analyses of induced pluripotent stem cell-derived DMD cardiomyocytes, which also showed development-dependent reductions (Willi et al. 2022). In both genotypes we found age-related changes in various elements of glucose metabolism, including its intracellular transport. This is likely due to an increase in the need for energy from glucose, which in mdx cerebra can be seen as early as at 10d, based on increased expression of complex I, II and V subunit-encoding genes (Fig. 6a), while respiration from pyruvate (complex I) and in complex II is not impaired. However, a much greater content of all ETC complexes was evident in the cerebrum of 10w mdx, suggesting a greater energy requirement there. Notably, the expression of major glycolysis genes (*HK3, Pfkp* and *Pkir*, Fig. 6a) was significantly lower in the 10w cerebra of both the mdx and control mice than in those of the 10d animals. This indicates a shift from glycolysis to oxidative phosphorylation that correlates with the maturation of the nervous system (Gallo 2020, 2024; Surin et al. 2013; Xavier et al. 2016). Our data, albeit limited to isolated mitochondrial respiration, indicate differences in energy metabolism between mdx and control animals. A previous study described a significant decrease in complex I-specific activity, not respiration, in the cerebra of 12-week-old mdx mice (Tuon et al. 2010). This finding does not correlate with our Western blot or OCR analyses. However, Tuon et al. (2010) analysed individual dystrophin-expressing regions while we used the entire cerebrum, where regional differences might be masked by higher amounts of ETC in other brain areas. It is also important to consider the complexity of the in vivo brain energy demand, where respiratory substrates include ketogenic compounds and fatty acids. Furthermore, significant increase in relevant transcripts in the cerebrum of mdx mice (clusters 1 and 4, Fig. 2c) correlating with increased protein levels in 10w mdx mice (Fig. 6c) support the increased energy metabolism. An investigation of earlier time points (between 10d and 10w) could provide additional insights.

When considering glucose metabolism, it is also important to remember the use of the glucose carbon skeleton for the synthesis of neurotransmitters such as glutamate, GABA and other amino acids, especially in the context of impaired GABAergic transmission in mdx brains (Rae et al. 2002).

The differences between the cerebra and cerebella might reflect varying degrees of neuroinflammation caused by inflammatory mediators crossing from the bloodstream to the brain at different rates depending on regional differences in BBB permeability. Our findings confirmed previous in situ enzyme histochemistry data on the presence of high CD39 and TNAP activity in the mouse brain vasculature (Langer et al. 2008) but revealed unaltered patterns of blood vessel-specific localization of these two enzymes in mdx mice (Fig. 14). These data suggest that the absence of any unfavourable changes in the vascularization of mdx brains does not align with the vascular and blood‒brain barrier integrity defects previously described (Nico et al. 2003, 2004). Those HRP permeability studies were conducted in 2-day-old and 18–20-month-old mdx mice (Nico et al. 2003), while structural defects were identified in embryos and in 4-week-old mice (Nico et al. 2004). Therefore, they should be present in 10w brains studied here, unless they are sex-specific (Dion-Albert et al. 2022), given that mice in the other studies were females.

The relatively intact BBB with absence of any signs of excessive vascularization or vascular leakage at 10w would limit inflammatory mediators permeability and would maintain normal nutrient and oxygen supply to the high energy-demanding brain tissue. It could also explain, to some extent, the lack of inflammation and microgliosis in 10w mdx brains (Fig. 14e). Interestingly, there were three upregulated (*Trem2, Cx3cl, and Ngf*) and two downregulated (*Il18* and *Bdnf*) transcripts in the dystrophic cerebra at 10d, i.e., before the onset of muscle inflammation. This might suggest that inflammation in the brain is an intrinsic and early symptom, just as it is in dystrophic muscle, where inflammation has been shown to precede dystrophic muscle damage (Chen et al. 2005; Haslett et al. 2002; Pescatori et al. 2007). Additionally, altered expression of NGF and BDNF can impact their specific functions (Ferraguti et al. 2023; Lombardi et al. 2017; Stansberry and Pierchala 2023; Zagrebelsky et al. 2020).

Two GO Molecular Function categories, namely Ubiquitination and Regulation of Intrinsic Apoptotic Signalling in Response to DNA Damage were enriched in 10w DEGs in the cerebella and cerebra. In inflammation, ubiquitination manages protein activity and stability (Humphries et al. 2018; Ohtake et al. 2016; Zagrebelsky et al. 2020), while the Regulation of Intrinsic Apoptotic Signalling Pathway in Response to DNA Damage can be secondary to inflammation.

Significant downregulation of L-kynurenine was observed in young cerebra and cerebella, which agrees with the findings of Copeland et al. (2022), indicating that young mdx mice may have lower levels of circulating kynurenic acid. Kynurenine metabolites have regulatory properties that could influence the inflammatory response in mdx brains. Moreover, low levels of L-kynurenine have been linked to neuropsychiatric abnormalities (Hafstad Solvang et al. 2019). However, modifications of the neuroprotective branch of the kynurenine pathway did not alleviate the mdx behavioural defects (Johnson et al. 2024). Further studies may explain the intricate interrelation between kynurenine, immunity, and brain functions in dystrophy.

Tuon et al. (2010) reported an increase in mitochondrial creatine kinase activity in the hippocampus, cortex, and striatum of dystrophic mice at 12 weeks. According to our transcriptomic data, creatine kinase was increased in dystrophic cerebra at 10d and 10w. This enzyme catalyses the reversible transfer of the phosphoryl group from phosphocreatine to ADP, which regenerates ATP (Losenkova et al. 2020). In dystrophic muscle, resveratrol treatment, which reduces serum creatine and reduces oxidative stress and induces mitophagy, improved dystrophic pathology (Sebori et al. 2018). However, in these muscles, mitophagy- and autophagy-related genes, including *Pink1, Becn1, and Map1lc3b*, were reduced (Sebori et al. 2018), and these genes were increased in 10-day-old dystrophic cerebra; *Mal13cb* was also increased in dystrophic cerebra at 10 weeks (Supplementary Table 9).

Our findings have therapeutic implications. Given that exon-skipping-driven restoration of dystrophin expression in the hippocampi, cerebella, and cortices of 6- to 8-week-old dystrophic mice significantly reduced some of their neuropsychiatric symptoms (Saoudi et al. 2023), these brain abnormalities appear to be amenable to treatment postnatally.

We have demonstrated that while abnormalities in the cerebrum arise early, those in the cerebellum, which are numerous, affect postnatal processes. Therefore, the Dp427 restoration described by Saoudi et al. (2023) should have a greater impact on the cerebellum, which was indeed the case. They found that fear-learning performance was particularly improved, which they connected to higher Dp427 levels in the cerebella. Another reason might be that the treatment was more effective in this later-affected region. Moreover, our findings indicate that behavioural and cognitive DMD defects may be reversed or at least improved by early postnatal treatments. This could involve dystrophin restoration but might also be achieved by targeting abnormalities downstream from the absence of dystrophin. GLUT1 downregulation at 10d presents an interesting target for treatment with a ketogenic diet and drugs enhancing Glut1 expression, as used in Glut1 deficiency syndrome (Tang et al. 2019). In fact, the beneficial effects of such diets in mdx mice have already been demonstrated (Fujikura et al. 2021; Radley-Crabb et al. 2011), with a recent study showing improvements in brain functions (Fausto et al. 2024).

The targeting of the unfolded protein response and mRNA splicing abnormalities is also gaining interest as therapeutic options, and both alterations are among the early abnormalities we identified. The unfolded protein response can be targeted by the ER stress inhibitor tauroursodeoxycholic acid (TUDCA) and the histone deacetylase (HDAC) inhibitor phenylbutyrate (4-PBA), which prevent protein misfolding and aggregation (Kubota et al. 2006; Lo et al. 2013). Both TUDCA and 4-PBA have been shown to improve cognitive functions in various paradigms (Kubota et al. 2006; Lo et al. 2013; Ricobaraza et al. 2009).

In conclusion, although extremely varied, molecular alterations underlying the neuropsychiatric alterations caused by the loss of full-length dystrophins in DMD patients could be targetable, especially in the postnatal period.

## Supplementary Information

Additional file 1.

Additional file 2.

Additional file 3.

## Data Availability

No datasets were generated or analysed during the current study.
