# Supplementary material for: Spatiotemporal diversity in molecular and functional abnormalities in the mdx dystrophic brain
Source: Mol Med. 2025 Mar 20;31:108. doi: 10.1186/s10020-025-01109-5 (PMC11924731; doi:10.1186/s10020-025-01109-5)
Supplement: Supplementary file 2 — Additional file 2. [file 10020_2025_1109_MOESM2_ESM.docx]

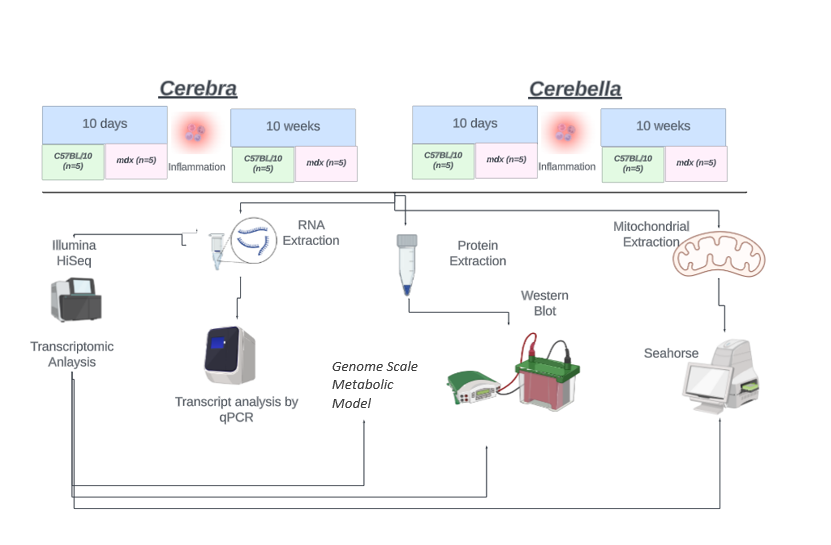


**Fig. S1 Schematic representation of the experimental design and analyses** *RNA, protein, and mitochondria were extracted from the cerebella and cerebra of 10-day-old and 10-week-old C57BL/10 and mdx mice (n=5 per group). RNA sequencing, quantitative polymerase chain reaction (qPCR), Western blotting, and oxygen consumption rate analysis were performed as described below [The image was made using BioRender and lucidchart. Last Accessed 21 / 11 / 2023].*


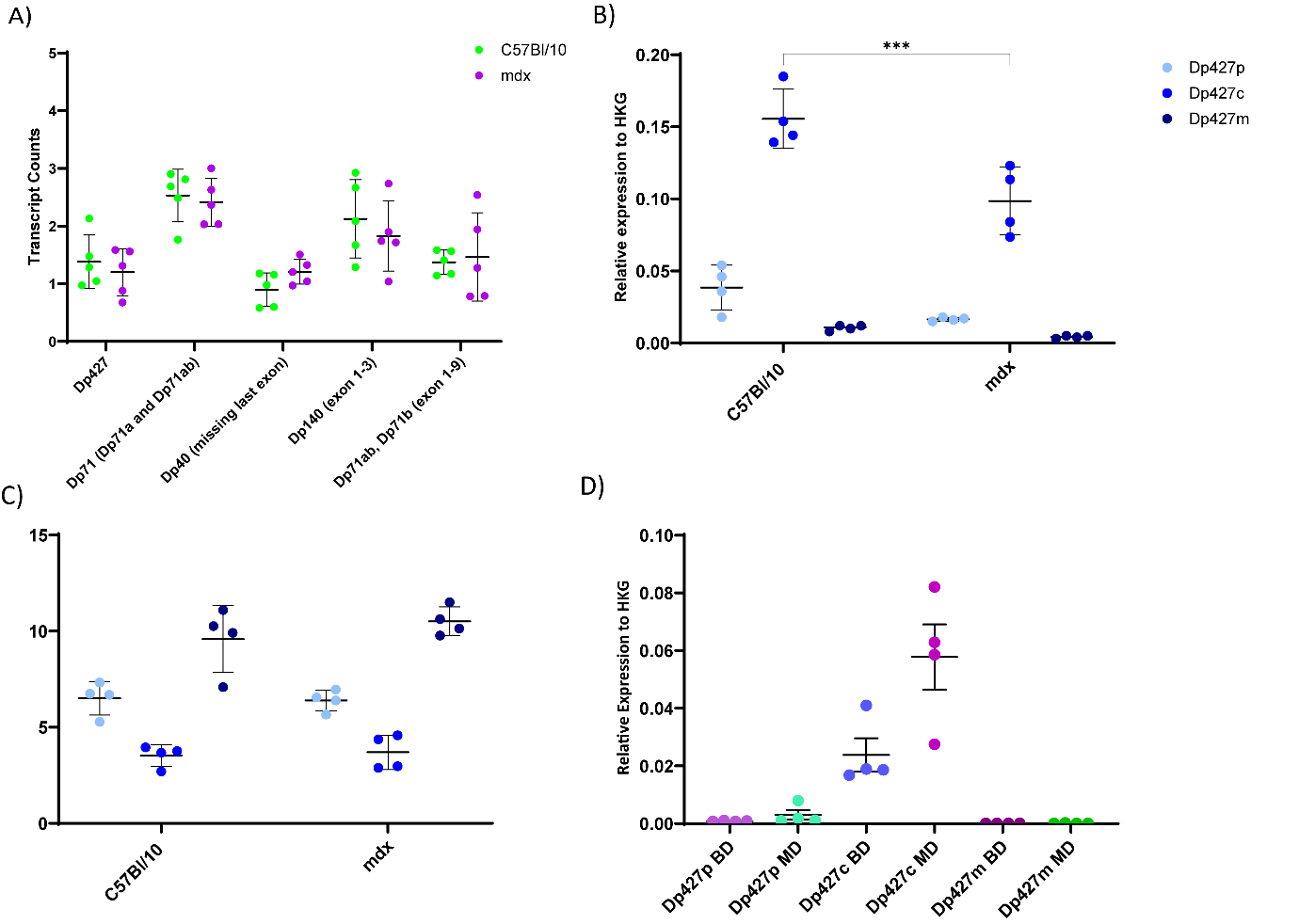


**Fig. S2 Expression levels of full-length dystrophin transcripts in cerebella and cerebra** *A) RNA-Seq dystrophin transcript expression data in* *10d mdx and C57Bl/10 cerebella. B) Expression level of full-length dystrophin transcripts in 10w mdx and C57Bl/10 cerebella, C) in 10d mdx and C57Bl/10 cerebella, D) in 10d mdx and C57Bl/10 cerebra. Normality of the data was determined using Shapiro-Wilks test. For B) and C) statistical significance was measured by 2 way ANOVA with Tukey’s post hoc test, for D) Kruska-Wallis test with Dunn’s multiple comparison as the data was not normally distributed; *** p-value < 0.001. BD = C57Bl/10 10 day old cerebra, MD = mdx 10 day old cerebra.*

**Fig. S3 Expression levels of shorter length dystrophin transcripts (Dp71 and Dp140)** **in cerebra and cerebella** *A) Expression level of Dp71 and Dp140 in 10w mdx and C57Bl/10 cerebra B) in 10d mdx and C57Bl/10 cerebra, C) in 10w mdx and C57Bl/10 cerebella D) and 10d mdx and C57Bl/10 cerebella. As the data was normally distributed confirmed by Shaprio-Wilks test, two-way ANOVA with Tukey’s posthoc test was employed to determine differences between the genotypes.*


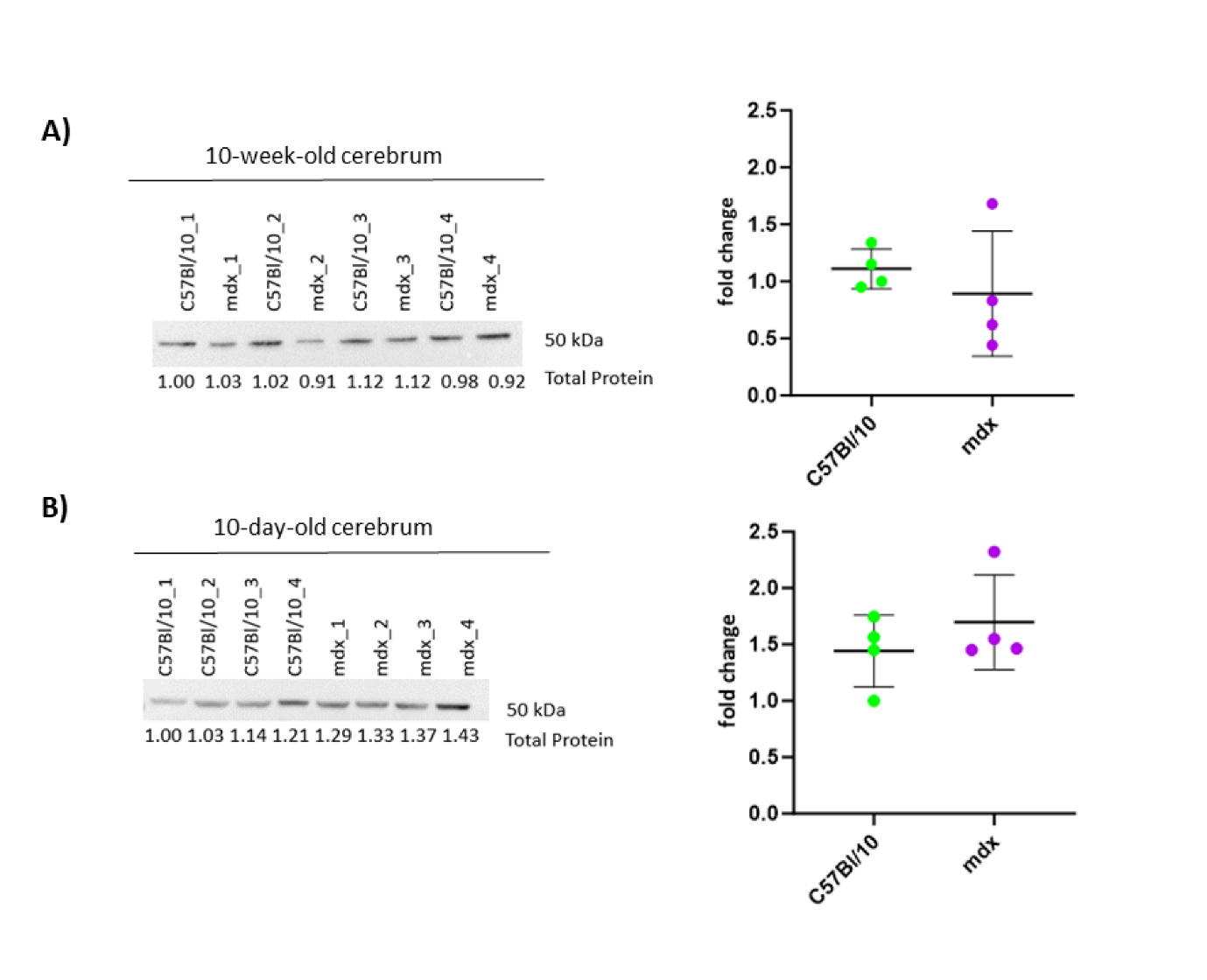


**Fig. S4: Expression level of GLUT3+14 in mdx and C57Bl/10 cerebra** *A) GLUT3+14 in 10w mdx and C57Bl/10 cerebra, B) in 10d mdx and C57Bl/10 cerebra. GLUT3+14 was detected by western blot and normalized to total protein in lines visualized with Ponceau S. Normalization factors are shown below the representative western blot images. The graph shows the mean values with SDs. Statistical significance was calculated using Student’s t test.*

**
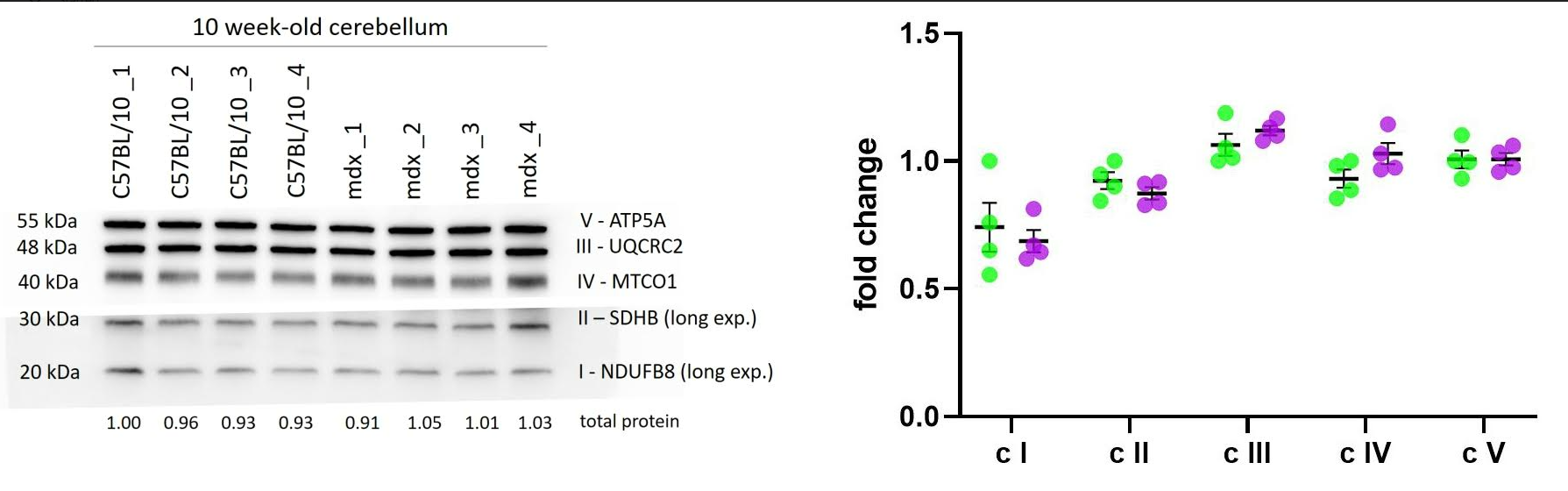
**

**Fig. S5** **Protein content of ETC subunits in 10w cerebella from C57Bl/10 and mdx mice** *ETC subunits: NDUFB8 (complex I; c I), SDHB (complex II; c II), UQCRC2 (complex III; c III), MTCO1 (complex IV; c IV) and ATP5A (complex V; c V) were detected by western blot procedure and normalized to total protein in lines visualized with Ponceau S. Normalization factors are shown under the representative western blot images. Charts present mean values with standard deviation. Statistical significance was calculated with Student t-test.*


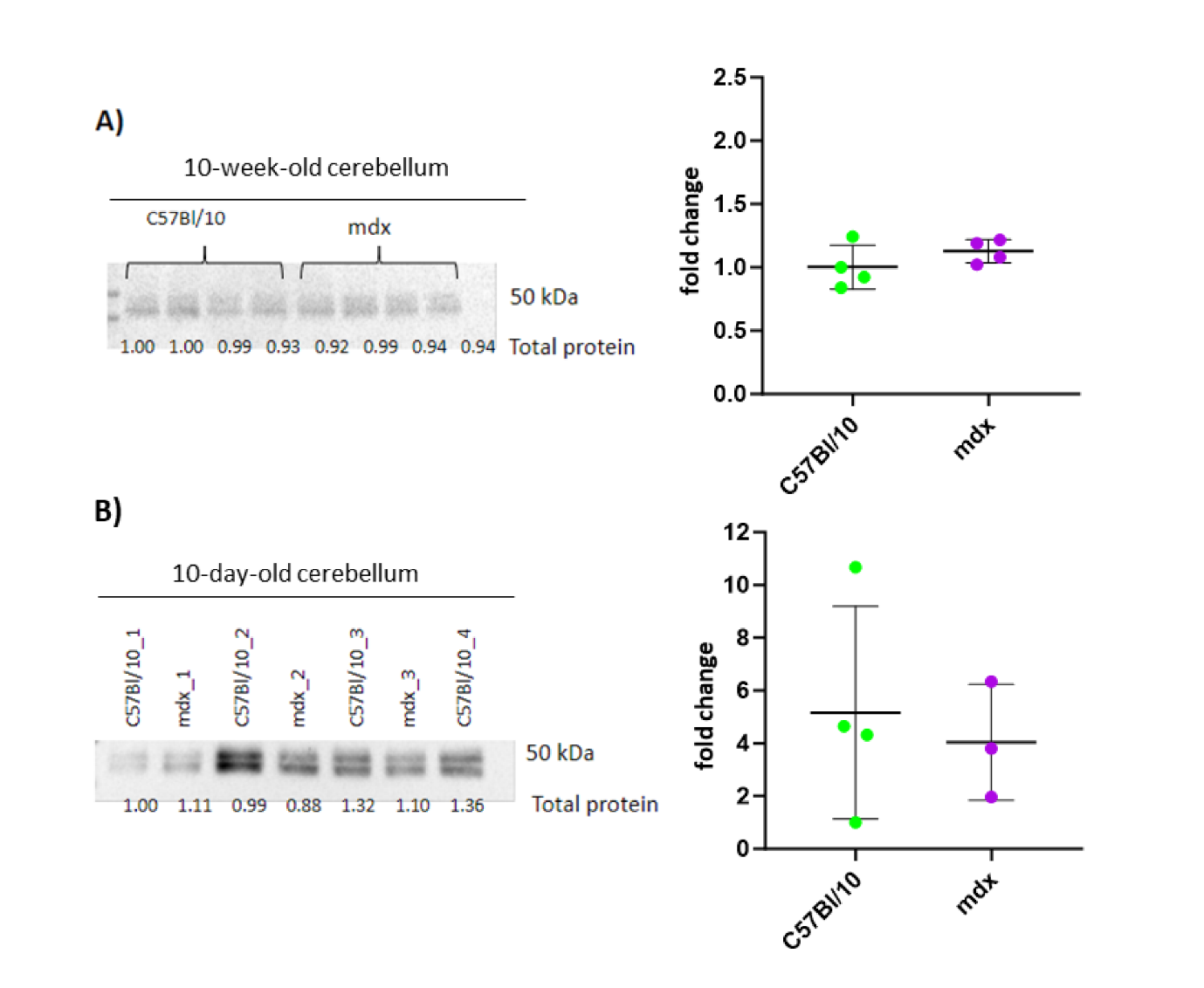


**Fig. S6 Expression level of GLUT1 in mdx and C57Bl/10 cerebella** *A) GLUT1in 10w mdx and C57Bl/10 cerebella, B) GLUT1 in 10d mdx and C57Bl/10 cerebella.* *GLUT1 was detected by western blot and normalized to total protein in lines visualized with Ponceau S. Normalization factors are shown below the representative western blot images. The graph shows the mean values with SDs. Statistical significance was calculated using Student’s t test.*


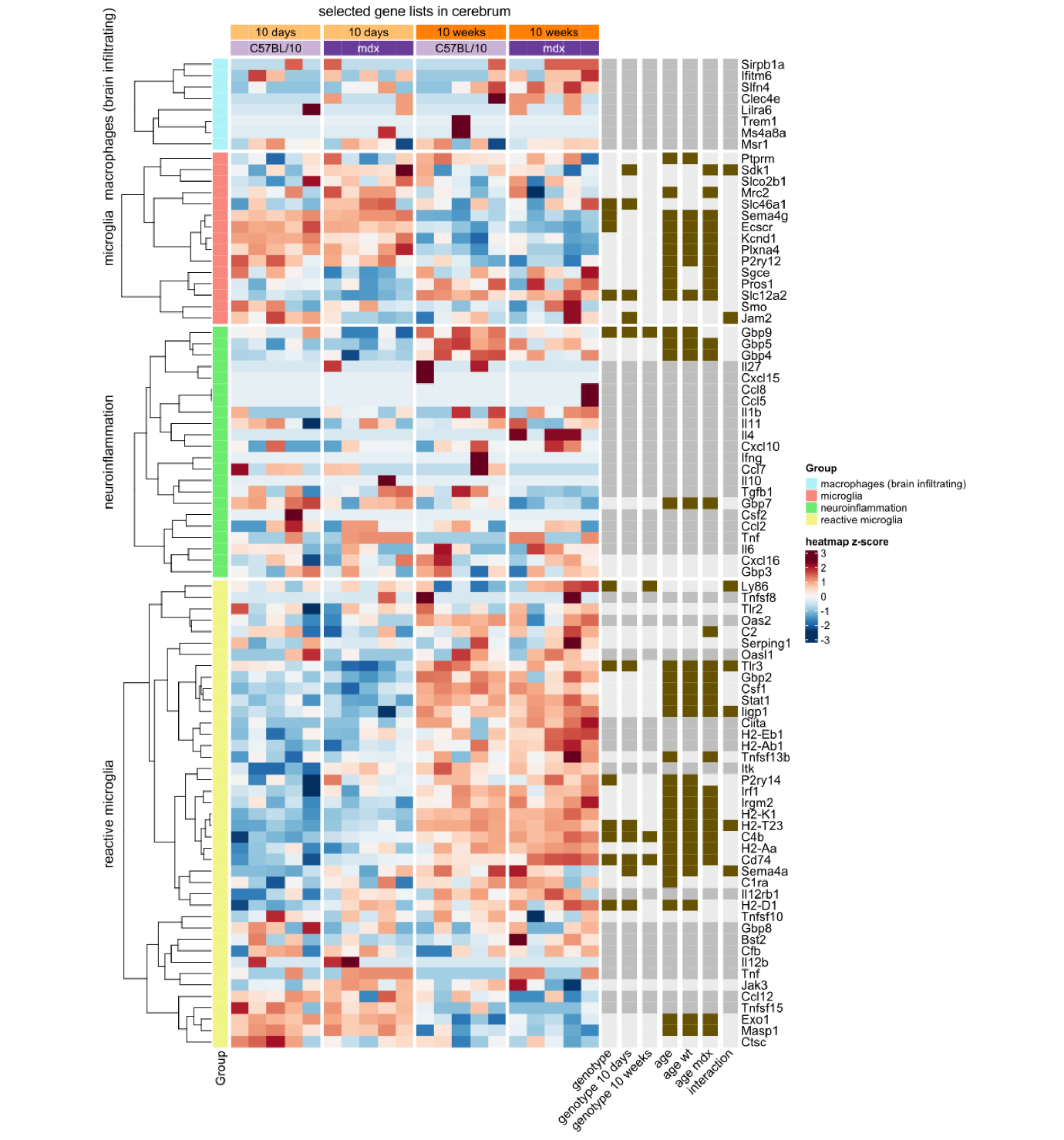


**Fig. S7 Heatmap of abundance of selected neuroinflammatory genes in cerebra.** *The intensity of the coloured rectangles represents transcript abundance levels. The presented level is proportional to the row z-score values (between darkest blue: −3 and darkest red: 3), as displayed on the bar above the heatmap image. To order rows (genes), hierarchical clustering was performed using correlation as a distance measure. The annotations in columns on the right of the heatmap highlight respectively whether the gene is differentially expressed (FDR < 10%) according to the global genotype effect and then in 10d and 10w mdx cerebra compared to age-matched controls or the global age affect and then in 10d C57Bl/10 cerebra compared to 10w C57Bl/10 cerebra and 10d mdx cerebra compared to 10w mdx cerebra.*


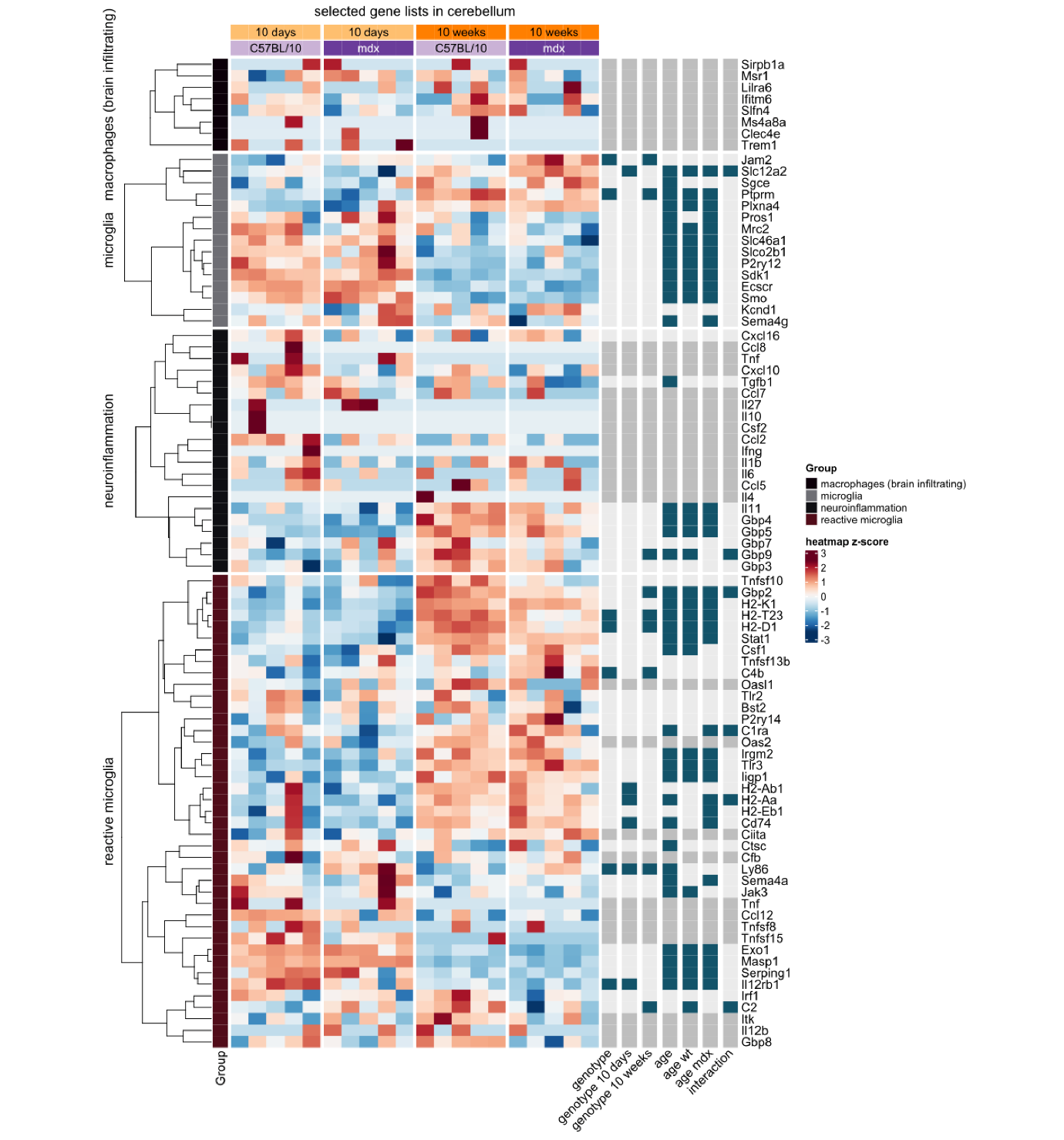


**Fig. S8 Heatmap of abundance of selected neuroinflammatory genes in cerebella.** *The intensity of the coloured rectangles represents transcript abundance levels. The presented level is proportional to the row z-score values (between darkest blue: −3 and darkest red: 3), as displayed on the bar above the heatmap image. To order rows (genes), hierarchical clustering was performed using correlation as a distance measure. The annotations in columns on the right of the heatmap highlight respectively whether the gene is differentially expressed (FDR < 10%) according to the global genotype effect and then in 10d and 10w mdx cerebella compared to age-matched controls or the global age affect and then in 10d C57Bl/10 cerebella compared to 10w C57Bl/10 cerebella and 10d mdx cerebella compared to 10w mdx cerebella.*

**Fig. S9 Expression levels of different P2X7 splice variants (P2X7a and P2X7b) in mdx and C57Bl/10 cerebra** *A) qPCR expression levels of P2X7a in 10d and 10w mdx and C57Bl/10 cerebra and B) P2X7k in 10d and 10w mdx and C57Bl/10 cerebra. Statistical significance was calculated with two-way ANOVA with Tukey's post hoc test.*


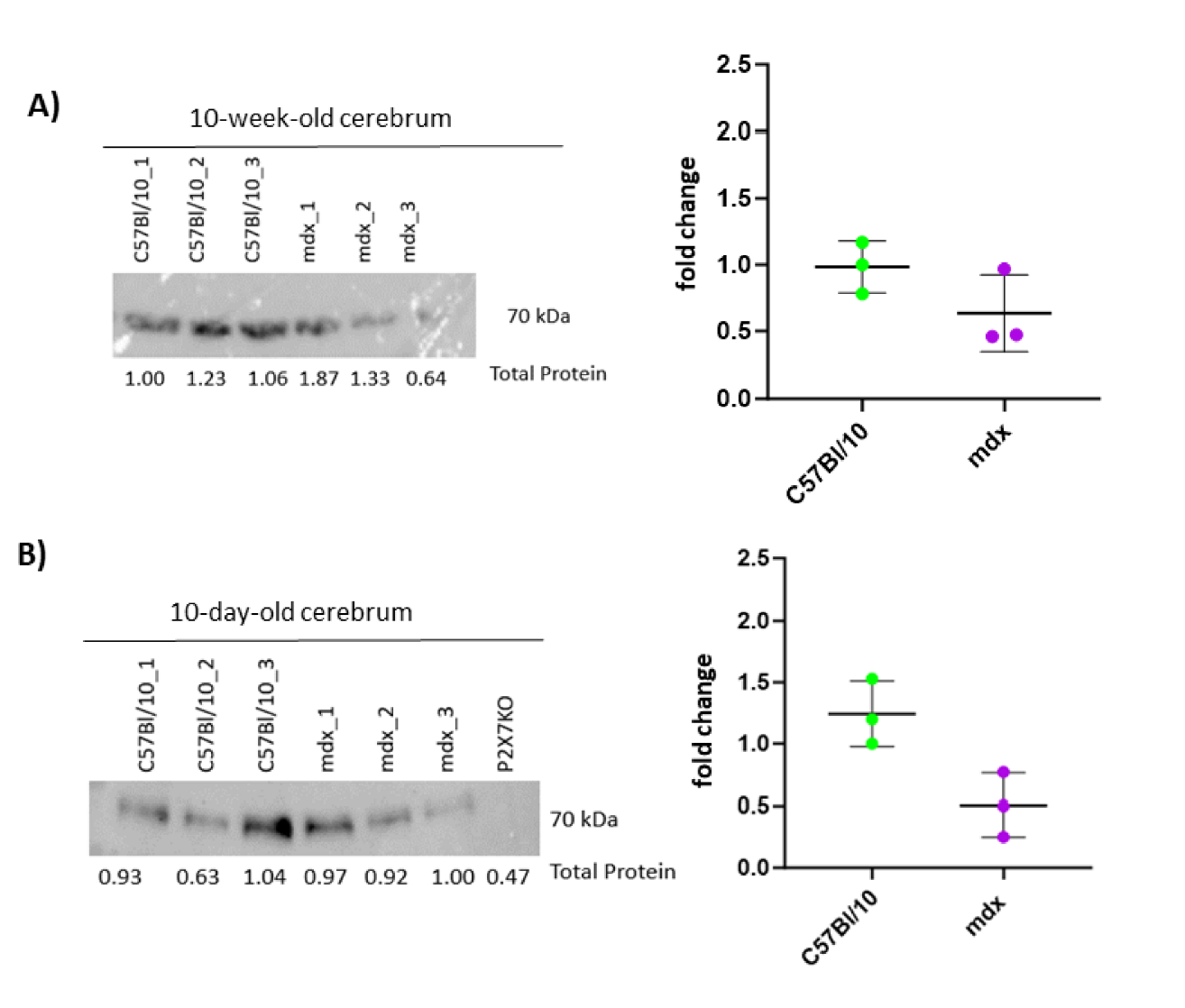


**Fig S10 Expression level of P2X7 in mdx and C57Bl/10 cerebra** *A) P2RX7 in 10w mdx and C57Bl/10 cerebra, B) P2RX7 in 10d mdx and C57Blk/10 cerebra.*  *P2RX7 was detected by western blot and normalized to total protein in lines visualized with Ponceau S. Normalization factors are shown below the representative western blot images. The graph shows the mean values with SDs. Statistical significance was calculated using Student’s t test.*


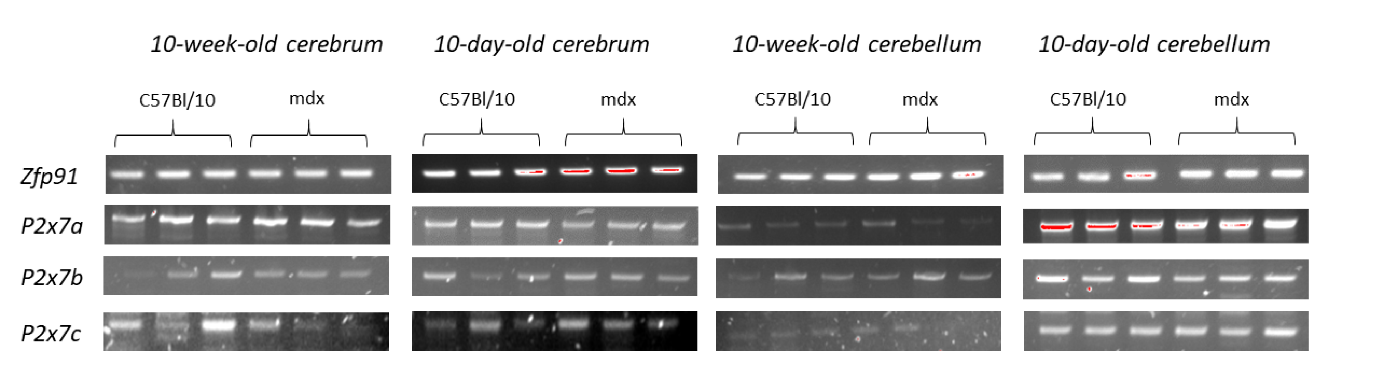


**Fig. S11 P2X7a,b,c 3’-nd splice variants,** *3’ splice variants of P2X7 (P2X7a, P2X7b, P2X7c) in 10-week-old and 10-day-old mdx and C57Bl/10 cerebra and cerebella at 10d and 10w. Zfp91 is shown in the first row as a loading control.*
